# Expression and promoter analysis of MEP pathway enzyme-encoding genes in *Pinus massoniana* Lamb

**DOI:** 10.7717/peerj.13266

**Published:** 2022-04-12

**Authors:** Peihuang Zhu, Yu Chen, Fan Wu, Miaojing Meng, Kongshu Ji

**Affiliations:** Co-Innovation Center for Sustainable Forestry in Southern China, Nanjing Forestry University, Nanjing, China

**Keywords:** *Pinus massoniana*, MEP pathway, Expression, Subcellular localization, Promoter

## Abstract

The methylerythritol phosphate (MEP) pathway provides the universal basic blocks for the biosynthesis of terpenoids and plays a critical role in the growth and development of higher plants. *Pinus massoniana* is the most valuable oleoresin producer tree with an extensive terrestrial range. It has the potential to produce more oleoresin with commercial value, while being resistant to pine wood nematode (PWN) disease. For this study, eleven MEP pathway associated enzyme-encoding genes and ten promoters were isolated from *P. massoniana*. Three *PmDXS* and two *PmHDR* existed as multi-copy genes, whereas the other six genes existed as single copies. All eleven of these MEP enzymes exhibited chloroplast localization with transient expression. Most of the MEP genes showed higher expression in the needles, while *PmDXS2*, *PmDXS3*, and *PmHDR1* had high expression in the roots. The expressions of a few MEP genes could be induced under exogenous elicitor conditions. The functional complementation in a *dxs*-mutant *Escherichia coli* strain showed the DXS enzymatic activities of the three *PmDXSs*. High throughput TAIL PCR was employed to obtain the upstream sequences of the genes encoding for enzymes in the MEP pathway, whereby abundant light responsive *cis*-elements and transcription factor (TF) binding sites were identified within the ten promoters. This study provides a theoretical basis for research on the functionality and transcriptional regulation of MEP enzymes, as well as a potential strategy for high-resin generation and improved genetic resistance in *P. massoniana*.

## Introduction

Terpenoids play a critical role in the support of plants against biotic stress, particularly for conifers. The oleoresin consists of complex mixtures of terpenoids, which are critical for their physical and chemical defenses against herbivores and pathogens (Alicandri et al., 2020; Celedon & Bohlmann, 2019). In the event of injuries, oleoresin exudes from resin ducts to seal wounds, while simultaneously limiting microbial invasion (Zulak & Bohlmann, 2010).

Isopentenyl diphosphate (IPP, C5) and dimethylallyl diphosphate (DMAPP, C5) are precursors for the biosynthesis of all terpenes in plants (Tetali, 2019). Two independent metabolic pathways (mevalonate (MVA) and methylerythritol phosphate (MEP)) generate IPP and DMAPP (Abbas et al., 2017; Pazouki & Niinemets, 2016). Subsequently, IPP and DMAPP generate dozens of different terpenoids when catalyzed by prenyltransferases (PTs) and terpenoid synthases (TPSs) (Alicandri et al., 2020; Celedon & Bohlmann, 2019). The MVA pathway is localized within the cytosol and enables the synthesis of sesquiterpenes and triterpenoids, while the MEP pathway is localized in the plastids and assists in the production of monoterpenoids and diterpenoids (Rodriguez-Concepcion & Boronat, 2015; Vranova, Coman & Gruissem, 2013).

**Figure 1:**
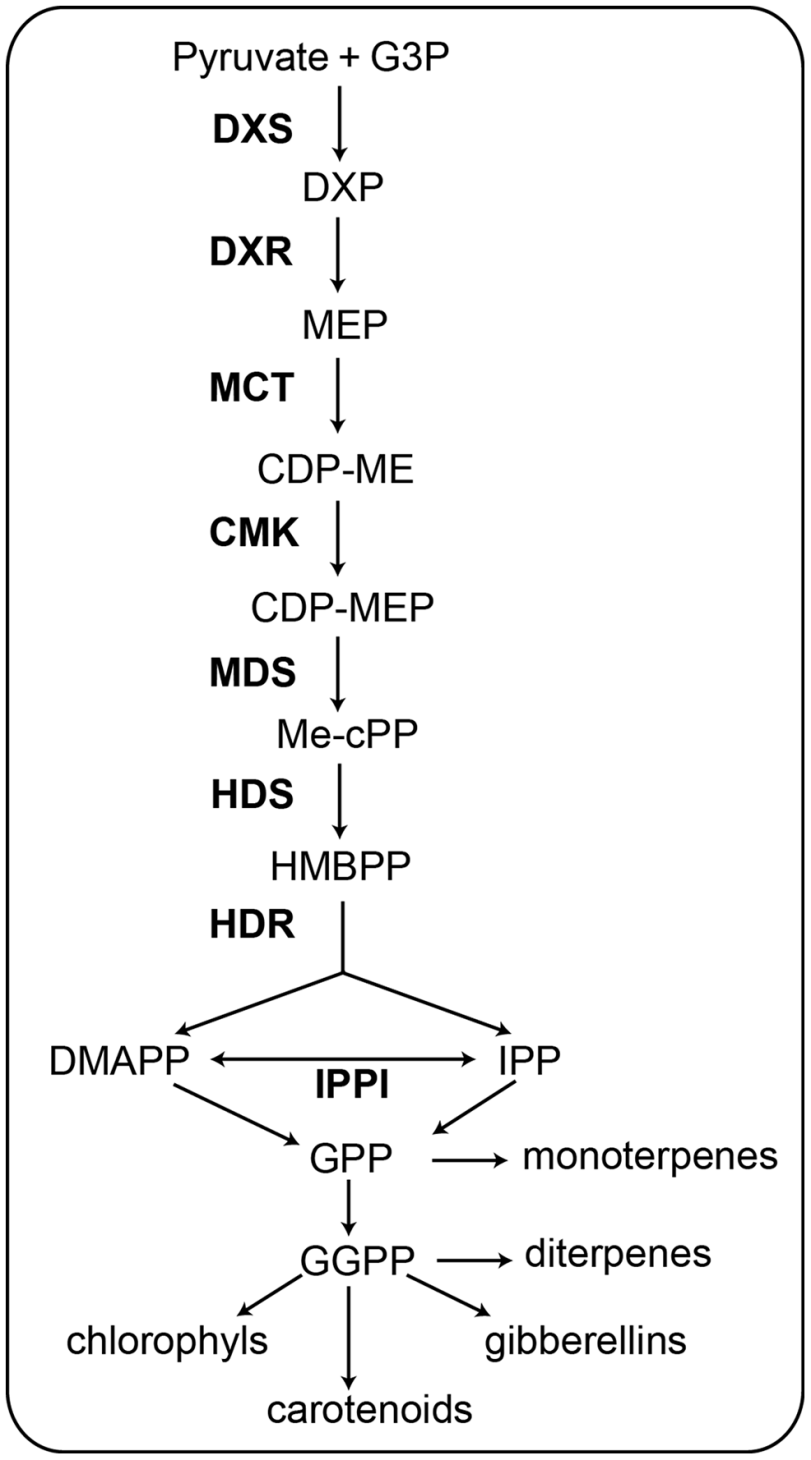
Schematic of the MEP pathway. All biosynthetic steps of the pathway are shown. The eight enzymes involved in the MEP pathway are shown in bold (Celedon & Bohlmann, 2019). G3P, glyceraldehyde 3-phosphate; DXS, 1-deoxy-d-xylulose 5-phosphate synthase; DXP, 1-deoxy-d-xylulose 5-phosphate; DXR, 1-deoxy-D-xylulose-5-phosphate reductoisomerase; MEP, 2-C-methyl-d-erythritol 4-phosphate; MCT, 2-C-methyl-D-erythritol 4-phosphate cytidylyltransferase; CDP-ME, -diphosphocytidyl-2-C-methyl-D-erythritol; CMK, 4-(cytidine 5′-diphospho)-2-C-methyl-D- erythritol kinase; CDP-MEP, 2-phosopho- 4-(cytidine 5′-diphospho)-2-C-methyl-D-erythritol; MDS, 2-C-methyl-D-erythritol 2,4-cyclodiphosphate synthase; Me-cPP, 2-C-methyl-d- erythritol 2,4-cyclodiphosphate; HDS, 4-hydroxy-3-methylbut-2-enyl diphosphate synthase; HMBPP, 4-hydroxy-3-methylbut-2-enyl diphosphate; HDR, 4-hydroxy-3-methylbut-2-enyl diphosphate reductase; IPPI, Isopentenyl diphosphate D-isomerase; IPP, isopentenyl diphosphate; DMAPP, dimethylallyl diphosphate; GPP, geranyl diphosphate; GGPP, geranylgeranyl diphosphate.

The MEP pathway contains seven successive reaction steps (Fig. 1). In the first step, pyruvate and glyceraldehyde-3-phosphate (G3P) are converted to 1-deoxy-d-xylulose 5-phosphate (DXP) when catalyzed by 1-deoxy-d-xylulose 5-phosphate synthase (DXS) (Phillips et al., 2007). In the second step, 1-deoxy-D-xylulose-5-phosphate reductoisomerase (DXR) catalyzes the transformation of DXP to 2-C-methyl-d-erythritol 4-phosphate (MEP) (Xu et al., 2019). In the third step, MEP is catalyzed by 2-C-methyl-D-erythritol 4-phosphate cytidylyltransferase (MCT) to produce 4-diphosphocytidyl-2-C-methyl-D-erythritol (CDP-ME). In the fourth step, CDP-ME is catalyzed by 4-(cytidine 5′-diphospho)-2-C-methyl-D- erythritol kinase (CMK) to produce 2-phosopho- 4-(cytidine 5′-diphospho)-2-C-methyl-D-erythritol (CDP-MEP). In the fifth step, CDP-MEP is catalyzed by 2-C-methyl-D-erythritol 2,4-cyclodiphosphate synthase (MDS) to produce 2-C-methyl-d- erythritol 2,4-cyclodiphosphate (Me-cPP). In the sixth step, Me-cPP is catalyzed by 4-hydroxy-3-methylbut-2-enyl diphosphate synthase (HDS) to generate 4-hydroxy-3-methylbut-2-enyl diphosphate (HMBPP). In the seventh and final step, HMBPP reductase (HDR) catalyzes HMBPP to yield isomeric IPP and DMAPP. The IPP and DMAPP can be interconverted *via* isopentenyl-diphosphate isomerase (IPPI) (Tholl, 2015).

Many of the MEP genes in higher plants have been isolated and their functions identified, including conifers (Celedon & Bohlmann, 2019; Vranova, Coman & Gruissem, 2013). The activities of all MEP enzymes have been confirmed for *Arabidopsis thaliana* (Vranova, Hirsch-Hoffmann & Gruissem, 2011). Three DXS isoforms were isolated and identified in *Picea. Abies* (Phillips et al., 2007), whereas two DXS isoforms, a DXR, and two HDR isoforms were cloned from *P. densiflora* (Kim et al., 2009). However, the researchers were primarily focused on the content of terpenoid products for commercial or ecological reasons, as well as the functional characterization and regulation of TPSs. However, the promoters of MEP pathway enzyme-encoding genes and their related transcription factors (TFs) have not, as yet, been well characterized in plants (Vranova, Coman & Gruissem, 2013). Previous studies have provided evidence to support the notion that TFs regulate the MEP pathway. For example, phytochrome interacting factors (PIFs) can regulate the expression of MEP genes in *A. thaliana* (Toledo-Ortiz, Huq & Rodriguez-Concepcion, 2010). Many conifers containing *Pinus massoniana* lack genomic reference data, which hinders the cloning of the promoters of MEP genes and transcriptional regulation studies in conifers.

*P. massoniana* is both an economically and ecologically important evergreen conifer in Southern China and is one of the most important forest tree species. It is known to be the largest oleoresin producer in the world, which supplies ~90% of the total oleoresin in China (Liu et al., 2015). Pine oleoresin is extensively used in pharmaceuticals, chemicals, biofuels, and other industries (Harvey, Wright & Quintana, 2010; Kelkar et al., 2006). However, millions of hectares of *P. massoniana* forests in China are seriously threatened by pine wilt disease (PWD) (Liu et al., 2021; Liu et al., 2015). PWD is a devastating forest disease caused by pine wood nematodes (PWNs), which are transmitted by insect vectors (*Monochamus* spp.) (Naves et al., 2007). PWN causes the loss of water transport function in the vascular systems of pine trees, which can quickly lead to wilting and death (Ye, 2019). As of 2020 PWN has killed ~19 million pine trees in China (Li et al., 2021a). Previous studies have revealed that certain terpenoids and their specific ratios might influence the behaviors of PWNs and their vector *M. alternatus* (Niu et al., 2012; Zhao et al., 2009; Zhao et al., 2007). Thus, the manipulation of the biosynthesis of terpenoids is a potential strategy for the breeding of PWN resistance in *P. massoniana*.

There have been few reports on the functional identification of MEP pathway genes for *P. massoniana* (*e.g*., a DXS isoform) (Li et al., 2021b), and no studies on the promoter analysis of MEP pathway enzyme-encoding genes. The two main goals of this work were to: (i) provide comprehensive sequences validated by Sanger sequencing of the MEP pathway genes and promoters in *P. massoniana*; and (ii) provide spatial and temporal insights into the MEP pathway for different tissues, and the responses to different signal transduction elicitors based on qPCR. This work lays the foundation for the elucidation of gene functions, the transcriptional regulation of the MEP pathway, as well as entire terpenoid biosynthesis networks for *P. massoniana*. Further, it provides a potential strategy for high-resin generation and improved genetic resistance for this species.

## Materials and Methods

### Plant materials and elicitor treatments

Seeds of *P. massoniana* were collected from the National *P. massoniana* Superior Variety Base of Xinyi, in Guangdong, China. Two-year-old *P. massoniana* plants were transplanted into pots containing a soil mixture (peat:perlite:vermiculite, 3:1:1 (v/v)) at 24 °C under a 16-h light/8-h dark photoperiod. Plants at similar growth stages were selected for different tissue collections and subsequent treatments.

Four elicitor treatments were applied (10 mM H_2_O_2_, 100 µM methyl jasmonate (MeJA), 500 μM ethephon (ETH), and 1 mM salicylic acid (SA)). It is important to note that MeJA does not dissolve easily in water; however, it is soluble in ethanol. Thus, MeJA was dissolved in 5 mL ethanol and then diluted with distilled water to volume in a 1 L volumetric flask. Finally, these solutions were sprayed onto the needles of the seedlings, which were exposed to the treatments for 0 h, 3 h, 12 h, and 24 h. Subsequently, the needles were collected for RNA extraction, where the samples treated with only the corresponding solvent were used as controls. Each treatment was repeated three times, which served as biological replicates. All collected samples were frozen in liquid nitrogen and stored at −80 °C for DNA and RNA extraction.

### Total RNA, gDNA extraction, and cDNA synthesis

The total RNA was extracted by the RNAprep Pure Kit (DP441; Tiangen Biotech, Beijing, China). A NanoDrop 2000 instrument (Thermo Fisher, Waltham, MA, USA) and 1.2% agarose gel electrophoresis were employed to detect the concentration, integrity and purity of the total RNA.

The cDNA (20 μL) was synthesized using a first-strand cDNA synthesis kit (11141; Yeasen Biotech, Shanghai, China) with 1,000 ng total RNA.

The genomic DNA (gDNA) was extracted using a Plant Genomic DNA Kit (DP320; Tiangen Biotech, Beijing, China). The gDNA concentration and purity were measured using a NanoDrop 2000 instrument, and the gDNA integrity was estimated *via* 1.2% agarose gel electrophoresis.

### Cloning of MEP pathway enzyme-encoding genes and sequence analysis

The ORF primers used for cloning the PCR are shown in Table S1. The PCR products were cloned into the pUC19 vector and Sanger sequencing was performed to verify the correction of the enzyme-encoding genes of the MEP pathway. The physicochemical properties of the MEP pathway enzyme proteins were analyzed using the ExPASy ProtParam online program (https://web.expasy.org/protparam/). Transmembrane domain analysis was performed using TMHMM Server version 2.0 (http://www.cbs.dtu.dk/services/TMHMM). Subcellular localization was predicted using the PSORT (https://psort.hgc.jp/) and Cell-PLoc version 2.0 programs (http://www.csbio.sjtu.edu.cn/bioinf/Cell-PLoc-2/). Multiple sequence alignment was performed using the Blastp (https://blast.ncbi.nlm.nih.gov/Blast.cgi) and DNAMAN programs. Conserved domain analyses were performed using the InterPro online program (http://www.ebi.ac.uk/interpro/).

### Subcellular localization

The vector construction and transient transformation were conducted as described previously (Zhu et al., 2021). Briefly, the CDS (Coding sequence) regions were inserted into a pBI121-GFP vector (35S::PmDXS1-GFP, *etc*.) that fused a green fluorescent protein (GFP). The primers used for vector construction are shown in Table S2. The fusion vectors were transformed into the *Agrobacterium tumefaciens* strain GV3101(pSoup-p19). Agrobacterium strains were grown in LB media for 36 h at 28 °C and then suspended in infiltration media (10 mM 2-(N-morpholino) ethanesulfonic acid (MES), 10 mM MgCl_2_, and 150 μM acetosyringone) for transient transformation. The suspension cells were infiltrated into the young leaves of 30- to 40-day-old *Nicotiana benthamiana*. Then the infiltrated tobacco plants were maintained under a 16 h light/8 h dark photoperiod at 22 °C for 48 h. The GFP and chloroplast fluorescence signals of the tobacco leaves were obtained using an LSM710 confocal laser scanning microscope (Zeiss, Jena, Germany).

### Isolation and sequence analysis of MEP pathway enzyme gene promoters

The promoter sequences were isolated using hiTAIL-PCR (high-efficiency thermal asymmetric interlaced PCR) from the genomic DNA of *P. massoniana*, as described by Liu & Chen (2007). The primers used for hiTAIL-PCR are shown in Table S3. Preamplification (20 μL), primary TAIL-PCR (50 μL), and secondary TAIL-PCR (50 μL) were performed as described by Liu & Chen (2007). The PCR products were cloned into the pUC19 vector and Sanger sequencing was performed. The structural analysis of the enzyme gene promoters in the MEP pathway were preformed using PLACE (https://www.dna.affrc.go.jp/PLACE/?action=newplace) and PlantCARE programs (http://bioinformatics.psb.ugent.be/webtools/plantcare/html/).

### Expression analysis of MEP pathway enzyme-encoding genes

Three biological replicates (plant samples) and three technical replicates (qPCR reactions) were used in expression analysis. In detail, for different tissues and elicitor treatments, corresponding samples were collected from three independent plants as biological replicates. In the qPCR assay, each reaction well was replicated three times (they contained the same cDNA template and primers), as technical replicates. The qPCR was conducted using a StepOne Plus instrument (Applied Biosystems, Waltham, MA, USA). The SYBR Green Mix (11184; Yeasen Biotech, Shanghai, China) and an internal control gene TUA (alpha-tubulin) were employed in the qPCR reactions (Zhu et al., 2019). The specific primers are shown in Table S4. Each PCR mixture (10 μL) consisted of 1 μL cDNA (20× dilution), 5 μL of SYBR Green Mix, 0.4 μL of each primer (10 μM), and 3.2 μL of ddH2O. The amplification was conducted as described previously (Zhu et al., 2021). The relative expression was computed by the 2^−ΔΔCt^ method (Livak & Schmittgen, 2001).

### Functional complementation

The EcAB4-2 strain of *Escherichia coli* was a *dxs* mutant, which was also MVA auxotrophy (Perez-Gil et al., 2012). This indicated that the EcAB4-2 strain survived only in the medium with exogenous MVA. The EcAB4-2 strain was used to test the functions of the *PmDXS1*, *PmDXS2*, and *PmDXS3* genes. The ORFs of the three *PmDXSs* were inserted into a pET-28a prokaryotic expression vector, with the primers used for vector construction shown in Table S2. These constructed vectors were used to transform the EcAB4-2 strain. The transformants were selected on Luria-Bertani (LB) solid medium with 25 μg/mL kanamycin, 15 μg/mL chloramphenicol, and 1 mM mevalonate (MVA).

## Results

### Cloning and sequence analyses of MEP pathway enzyme-encoding genes

**Figure 2:**
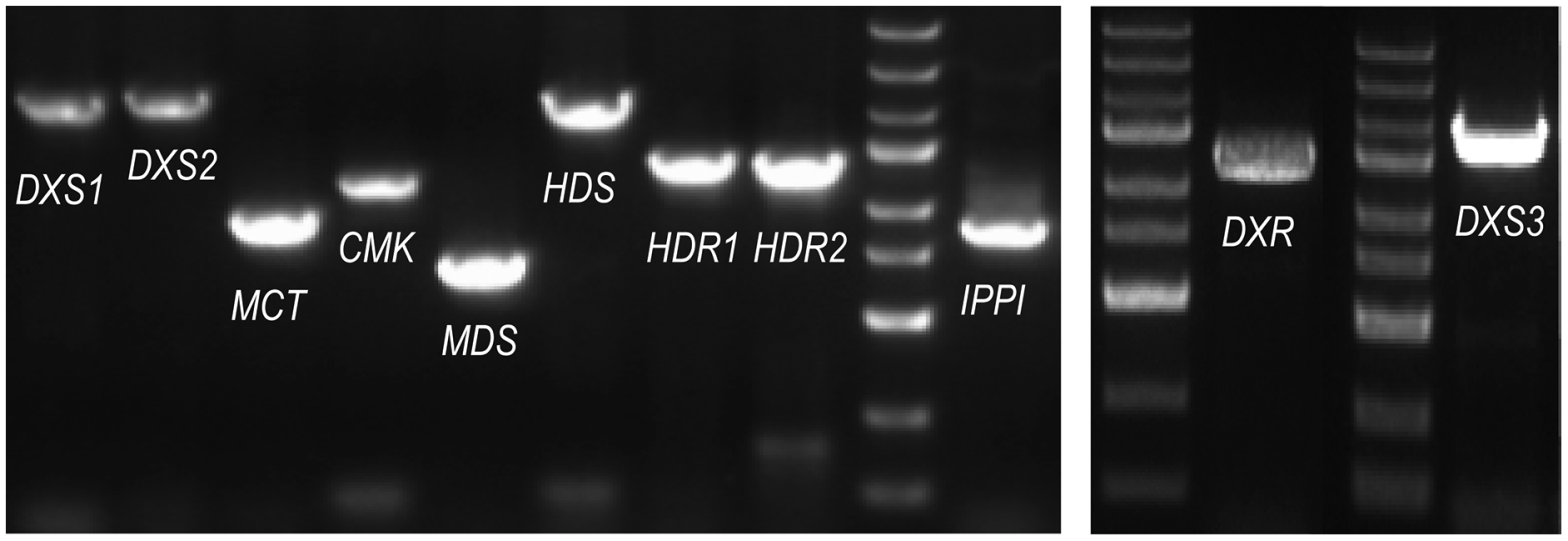
PCR gel bands for MEP pathway enzyme-encoding genes in *P. massoniana*. M represents the DNA ladder, from top to bottom, 5,000 bp, 3,000 bp, 2,000 bp, 1,500 bp (bright), 1,000 bp, 750 bp, 500 bp (bright), 250 bp, and 100 bp. The length of 11 ORF (open reading frame) from left to right, is, 2,154 bp (*PmDXS1*), 2,217 bp (*PmDXS2*), 954 bp (*PmMCT*), 1,218 bp (*PmCMK*), 726 bp (*PmMDS*), 2,232 bp (*PmHDS*), 1,464 bp (*PmHDR1*), 1,458 bp (*PmHDR2*), 939 bp (*PmIPPI*), 1,440 bp (*PmDXR*) and 2,223 bp (*PmDXS3*) respectively.

The ORF (open reading frame) sequences of the MEP pathway enzyme genes were obtained using the online program ORFfinder (https://www.ncbi.nlm.nih.gov/orffinder/) based on unassembled full-length transcripts from the Iso-seq data. PCR and Sanger sequencing verified the ORFs of eleven enzyme genes in the MEP pathway (PmDXS1, PmDXS2, PmDXS3, PmDXR, PmMCT, PmCMK, PmMDS, PmHDS, PmHDR1, PmHDR2, and PmIPPI) comprised of 2,154 bp, 2,217 bp, 2,223 bp, 1,440 bp, 954 bp, 1,218 bp, 726 bp, 2,232 bp, 1,464 bp, 1,458 bp, and 939 bp, respectively (Fig. 2). Eleven CDSs (Coding sequences) of MEP pathway enzyme-encoding genes from *P. massoniana* were submitted to the NCBI (accession number MW892440–MW892450) and are displayed in Table S1. The amino acid lengths, molecular weights, isoelectric points (pI), and transmembrane domains are shown in Table 1.

**Table 1:** Basic data for eleven MEP pathway genes in *P. massoniana*.

Gene	GenBank	ORF(bp)	Amino acids (aa)	Molecular weight (KDa)	Hydrophobicity/hydrophilicity	Transmembrane domain	pI
*PmDXS1*	MW892440	2154	717	77.16	Hydrophilicity	None	6.40
*PmDXS2*	MW892441	2217	738	79.22	Hydrophilicity	None	8.38
*PmDXS3*	MW892442	2223	740	79.34	Hydrophilicity	None	8.54
*PmDXR*	MW892443	1440	479	52.08	Hydrophilicity	None	6.29
*PmMCT*	MW892444	954	317	35.23	Hydrophilicity	None	8.98
*PmCMK*	MW892445	1218	405	44.34	Hydrophilicity	None	6.71
*PmMDS*	MW892446	726	241	25.75	Hydrophobicity	None	8.54
*PmHDS*	MW892447	2232	743	82.60	Hydrophilicity	None	6.35
*PmHDR1*	MW892448	1464	487	54.87	Hydrophilicity	None	5.93
*PmHDR2*	MW892449	1458	485	54.55	Hydrophilicity	None	5.98
*PmIPPI*	MW892450	939	312	35.19	Hydrophilicity	None	5.95

**Figure 3:**
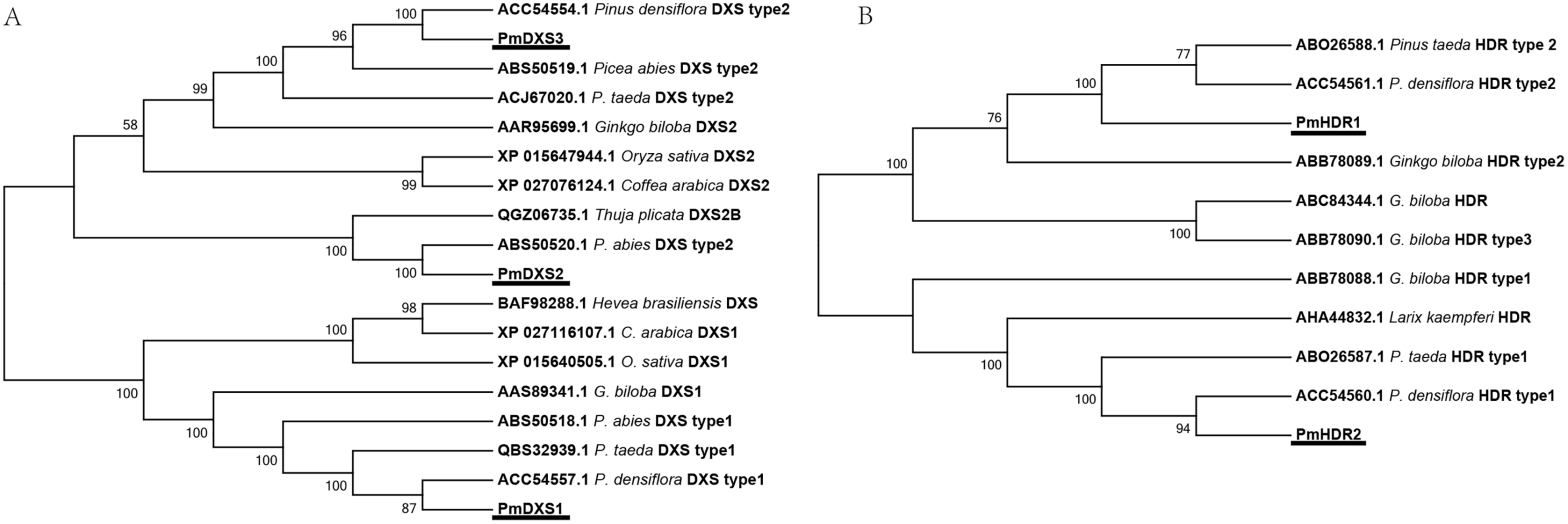
Phylogenetic trees of DXSs and HDRs. The tree was constructed using the MEGA program with a neighbor-joining (NJ) algorithm. (A) Phylogenetic trees of DXSs; (B) phylogenetic trees of HDRs. The underlined text represents the names of DXSs or HDRs proteins in *P. massoniana*, the text in italics represents the Latin names of other plants, and numbers represent the GenBank ID of homologous proteins from other plants.

The results of multiple alignments in the NCBI database revealed that the amino acid sequences of the eleven MEP genes from *P. massoniana* possessed a high homology with other Pinaceae plants. The results of multiple alignments with other plants (except for Pinaceae) revealed that the identities ranged from 59.37% to 88.97%, in which PmDXS1, PmDXR, and PmHDS shared higher identities (ranging from 73.88% to 88.97%), and where PmCMK showed the lowest identity (ranging from 59.37% to 74.61%) (Fig. S1). Furthermore, the alignment results suggested that PmDXS1 and PmDXS2 belonged to type II DXS, and PmDXS1 belonged to type I DXS. Similarly, between the two HDR homologs, PmHDR1 and PmHDR2 belonged to type II and type I, respectively. The results of phylogenetic grouping clearly showed three DXS (Fig. 3A) and two HDR (Fig. 3B) homologous proteins clustered into independent clades.

**Figure 4:**
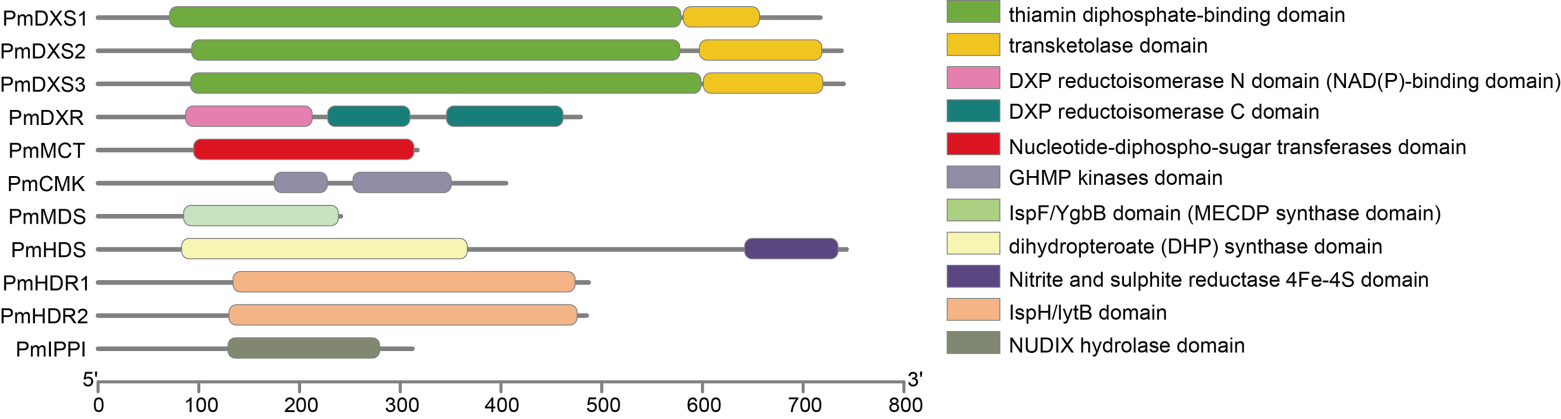
Conserved domains of MEP pathway enzymes in *P. massoniana*. The conserved domain was analyzed using the online InterPro program.

Conserved domain analyses are shown as a cartoon in Fig. 4. The results indicated that PmDXS1, PmDXS2, and PmDXS3 proteins had highly similar domains, including a thiamin diphosphate-binding domain at the N-terminal, and a transketolase domain at the C-terminal. The PmDXR protein contained three reductoisomerase domains at 87-214aa, 228-311aa, and 343-463aa, in which the first reductoisomerase domain at the N-terminal was also considered a NAD(P)-binding domain. A nucleotide-diphospho-sugar transferase domain was found at the C-terminal of the PmMCT protein. The PmCMK protein contained two GHMP kinase domains at 175-229aa and 253-353aa. A MECDP synthase domain (also known as the IspF/YgbB domain), was found at the C-terminal of the PmMDS protein. The PmHDS protein showed a dihydropteroate (DHP) synthase domain at the N-terminal and a nitrite and sulphite reductase 4Fe-4S domain at the C-terminal. Both PmHDR1 and PmHDR2 proteins revealed a LytB/IspH domain. The PmIPPI protein contained a NUDIX hydrolase domain at 129-281aa. The conserved domains between the MEP pathway enzyme proteins were crucial for their specific biological functions.

### Eleven MEP pathway enzymes in *P. massoniana* target the *N. benthamiana* chloroplast

**Figure 5:**
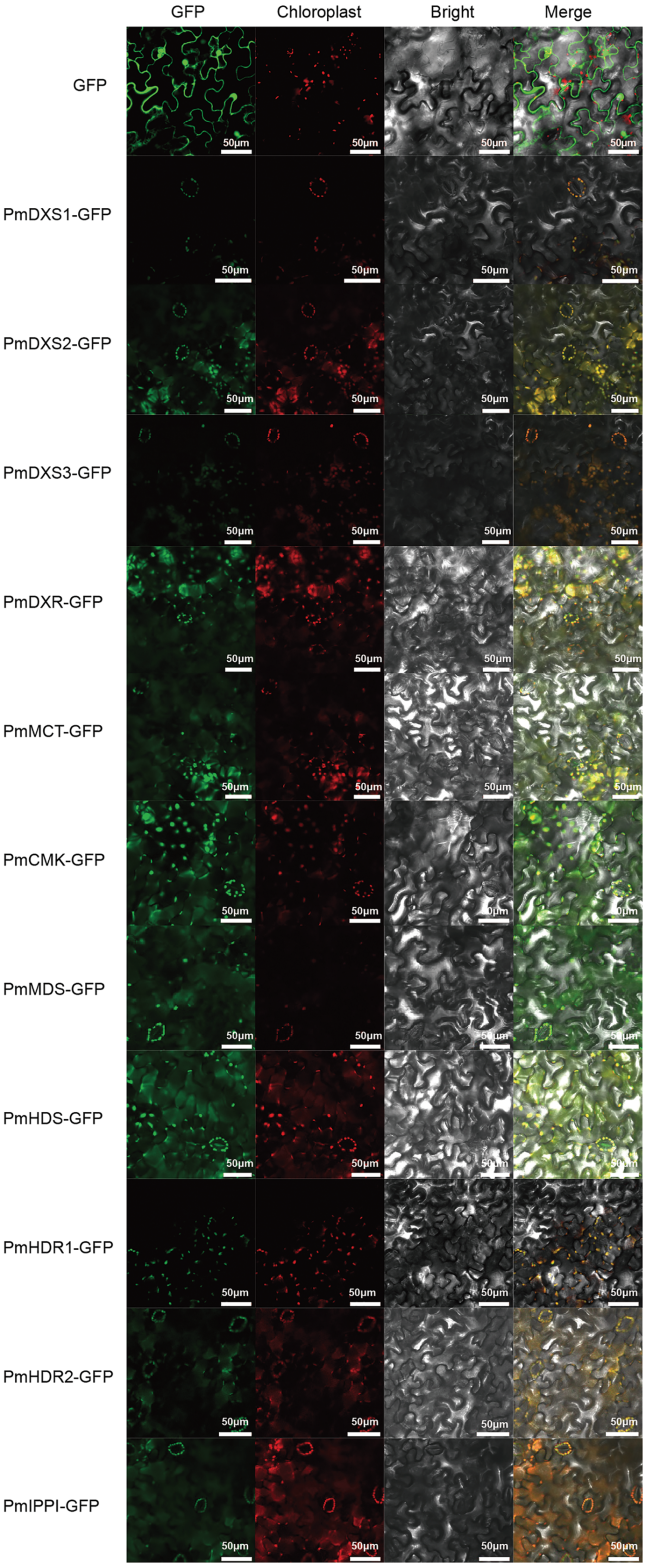
Results of subcellular localization assays of eleven MEP enzymes in *Nicotiana benthamiana*. The ORFs of MEP pathway enzyme-encoding genes from *P. massoniana* were fused in frame to GFP and transient transformed to *N. benthamiana* plants *via A. tumefaciens*. Fluorescence images were obtained using a LSM710 confocal laser scanning microscope. All scale bars represent 50 μm. GFP, green fluorescence protein images; Chloroplast, chloroplast auto-fluorescence images; Bright, bright field images; Merge, merged images of GFP, chloroplast and bright.

PSORT and Cell-PLoc were used for the prediction of subcellular localization, where according to both methods, the eleven MEP pathway enzyme proteins had the same predicted localizations within the chloroplast (Table 2). Furthermore, a transient expression experiment was performed to further explore the subcellular localization characteristics of the eleven MEP pathway enzyme proteins. The GFP fluorescent signals of the eleven MEP pathway enzyme proteins were observed to overlap with the chloroplast red fluorescence signals. This indicated that the subcellular localizations of the eleven MEP pathway enzyme proteins were targeted to the chloroplast (Fig. 5).

**Table 2:** Subcellular localization prediction.

Protein	Cell-PLoc	PSORT
PmDXS1	Chloroplast	chlo: 12, mito: 2
PmDXS2	Chloroplast	chlo: 10, mito: 4
PmDXS3	Chloroplast	chlo: 8, cyto: 4, mito: 1, plas: 1
PmDXR	Chloroplast	chlo: 9, cyto: 3, plas: 2
PmMCT	Chloroplast	chlo: 14
PmCMK	Chloroplast	chlo: 12, nucl: 2
PmMDS	Chloroplast	chlo: 11, mito: 1, vacu: 1, E.R.: 1
PmHDS	Chloroplast	chlo: 11, cyto: 1, mito: 1, cysk: 1
PmHDR1	Chloroplast	chlo: 9, cyto: 2, pero: 2, vacu: 1
PmHDR2	Chloroplast	chlo: 11, cyto: 3
PmIPPI	Chloroplast	chlo: 8, mito: 2.5, cyto_mito: 2, nucl: 1, plas: 1, extr: 1

### Expression patterns of MEP pathway enzyme-encoding genes in different tissues

**Figure 6:**
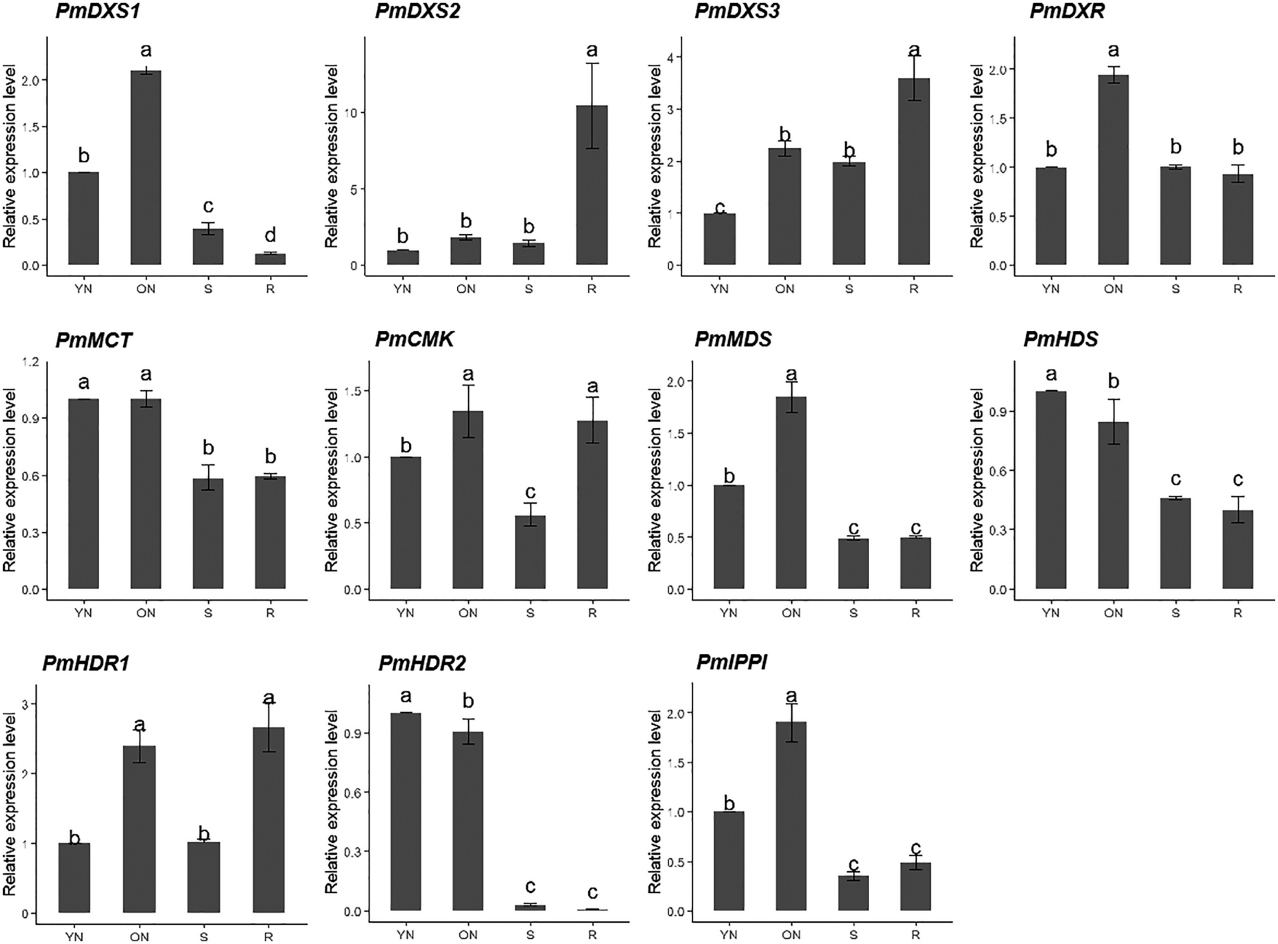
Expression patterns of eleven MEP pathway enzyme-encoding genes in different tissues. YN, young needles; ON, old needles; S, stems; R, roots. Different letters on the bar chart indicate significant differences between tissues at *p* = 0.05.

The qPCR was performed to investigate the tissue-specific expressions of the eleven MEP genes in young needles, old needles, stems, and roots of *P. massoniana*. Tissue-specific expression patterns suggested that most of the MEP genes had higher expression levels in the needles. PmDXS1, PmDXR, PmMCT, PmCMK, PmMDS, and PmIPPI showed the highest transcript levels in old needles, whereas PmHDS and PmHDR2 revealed the highest transcript levels in young needles (Fig. 6). Interestingly, three DXSs and two HDRs homologous genes had different expression patterns. PmDXS2 and PmDXS3 had significantly high transcript levels in the roots, which was quite different from PmDXS1. In particular, the expression of PmDXS2 in the roots were 10.45-fold, 5.84-fold, and 7.34-fold higher in young needles, old needles, and stems, respectively. PmHDR1 showed the highest expression levels in roots, while PmHDR2 showed quite low expression levels in stems and roots. The expression of PmHDR2 in young needles was 31.58- and 176.47-fold higher than that in stems and roots, respectively.

**Figure 7:**
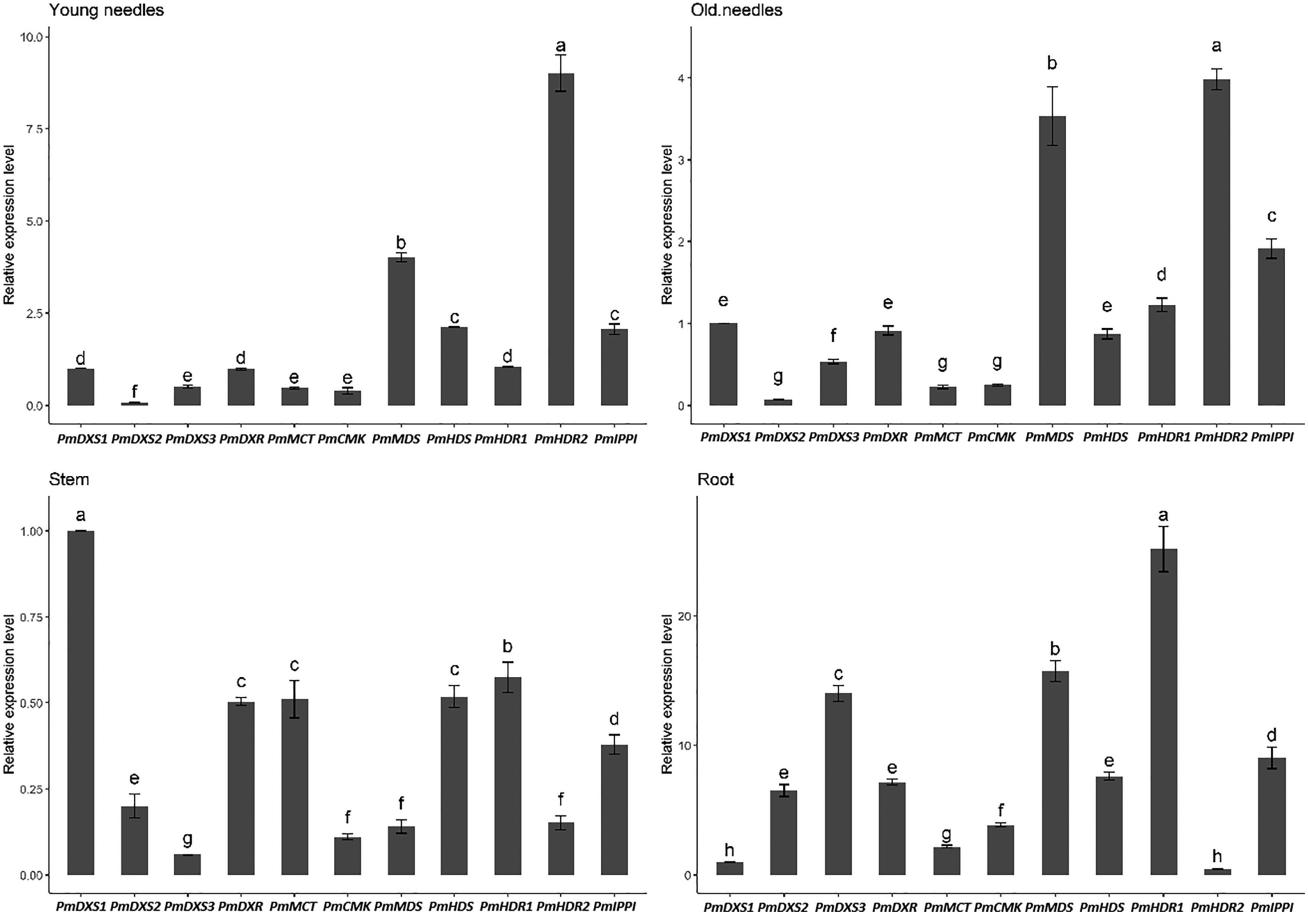
Expression patterns of eleven MEP pathway enzyme-encoding genes in the same tissue. Different letters on the bar chart indicate significant differences between genes at *p* = 0.05.

The results of the eleven MEP gene expression levels in the same tissue revealed that most had similar patterns in both young and old needles (Fig. 7). PmHDR2 showed the highest transcript level in the needles, followed by PmMDS and PmDXS2. PmDXS1 revealed the highest transcript level in the stems, followed by PmHDR1 and PmDXS2. Finally, PmHDR1 had the highest expression level in the roots, with PmMDS being the second highest, and PmHDR2 the lowest.

### Expression patterns of MEP pathway enzyme-encoding genes under different elicitor treatments

**Figure 8:**
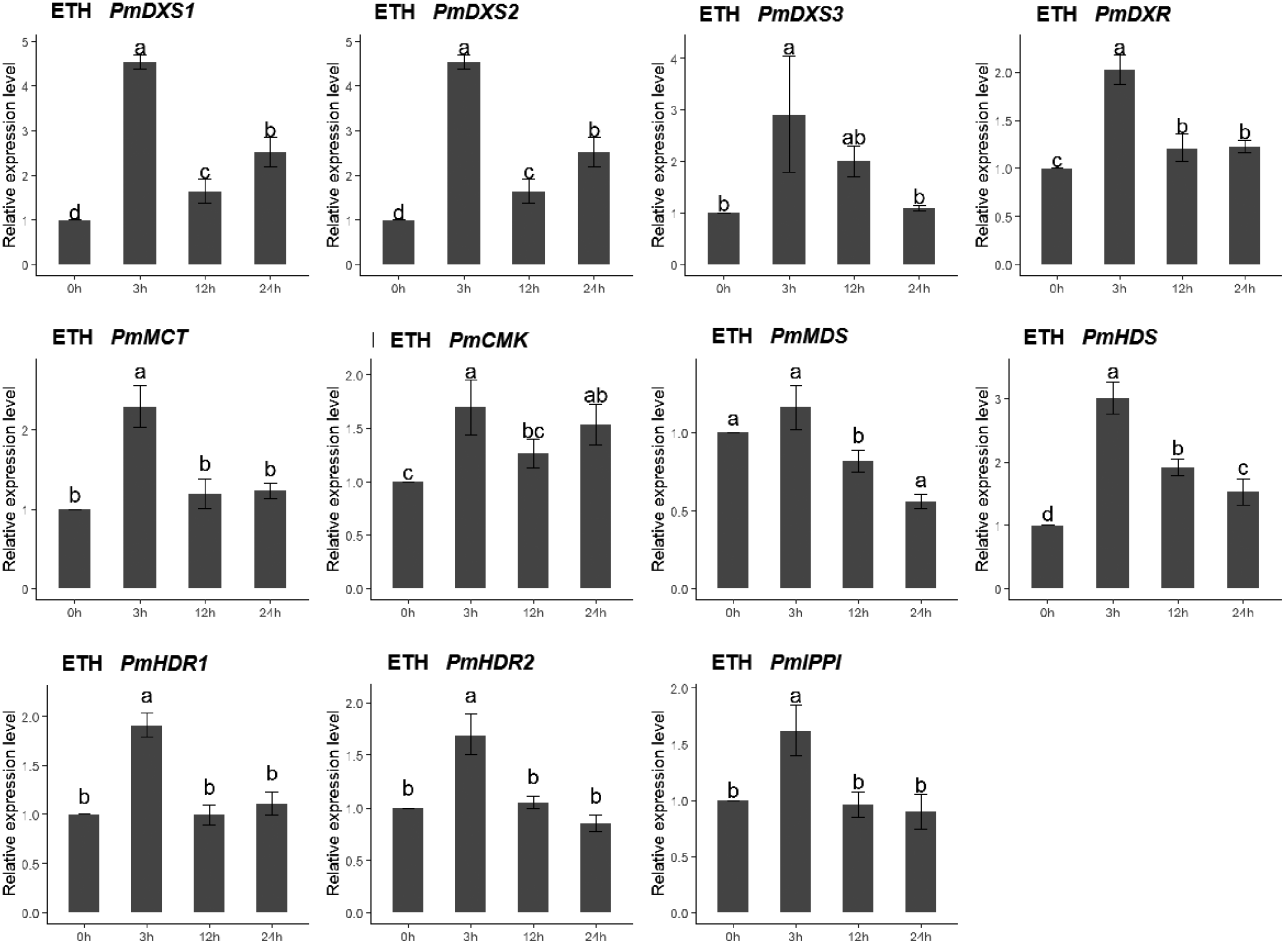
Expression patterns of eleven MEP pathway enzyme-encoding genes under the ETH treatment (500 μM). The EHT solution was sprayed onto the needles of the seedlings. Different letters on the bar chart indicate significant differences between treatment times at *p* = 0.05.

The qPCR was performed to investigate the expression patterns of the eleven MEP genes under ETH, MeJA, SA, and H_2_O_2_ elicitor treatments. For the ethephon (ETH) treatment, except for PmMDS, the other ten MEP genes had significantly high expression levels at 3 h (1.62- to 4.54-fold). However, the expression of PmMDS was substantially downregulated at 12 h and 24 h, particularly at 24 h (0.56-fold) (Fig. 8).

**Figure 9:**
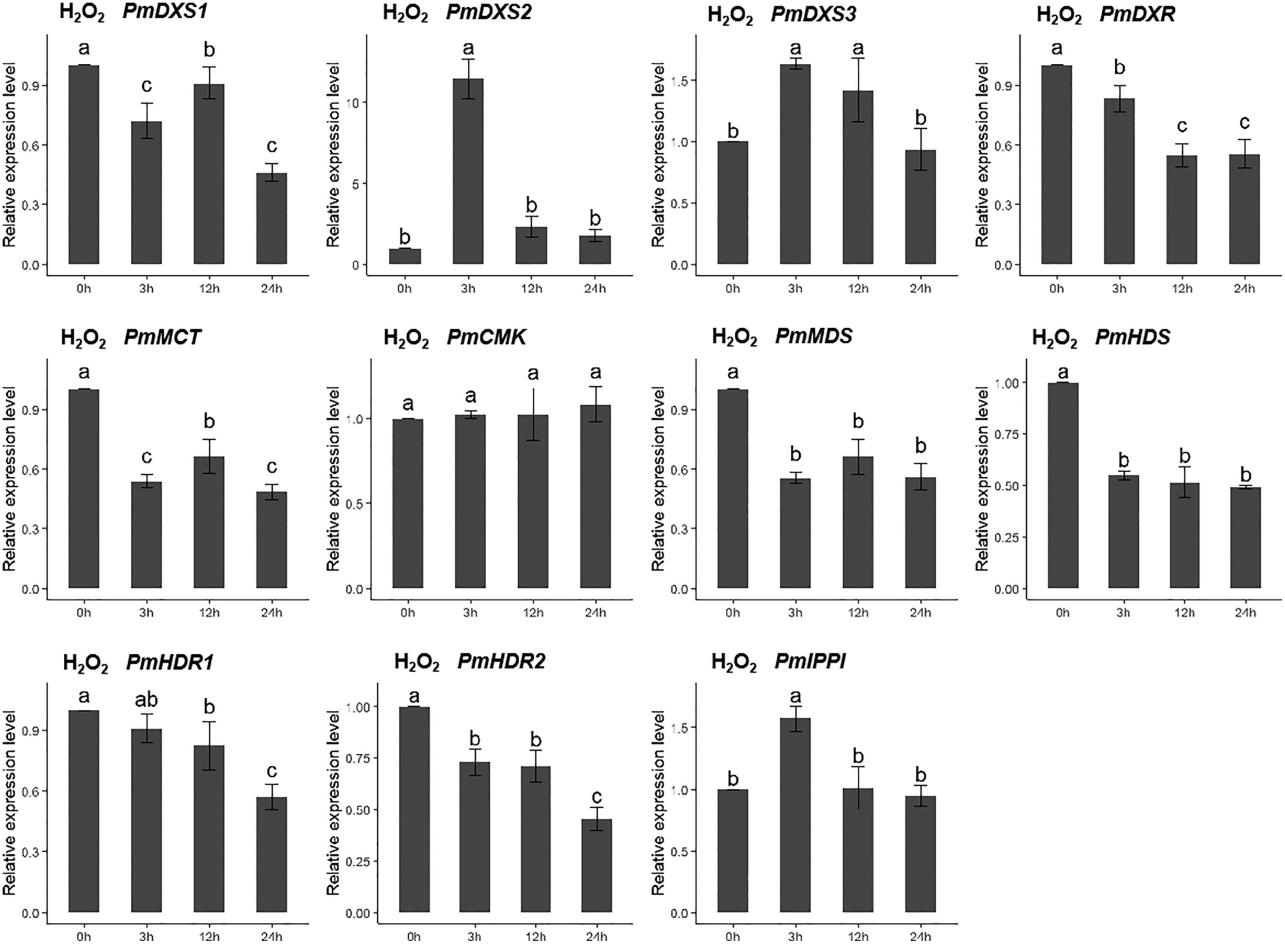
Expression patterns of eleven MEP pathway enzyme-encoding genes under the H_2_O_2_ treatment (10 mM). The H_2_O_2_ solution was sprayed onto the needles of the seedlings. Different letters on the bar chart indicate significant differences between treatment times at *p* = 0.05.

For the H_2_O_2_ treatment, the PmCMK expression level showed no significant change across all time points. The expressions of PmDXS1, PmDXR, PmMCT, PmMDS, PmHDS, and PmHDRs were considerably downregulated following the H_2_O_2_ treatment. The expression levels of PmDXS2, PmDXS3, and PmIPPI were strongly upregulated at 3 h (11.48-, 1.64-, and 1.69-fold, respectively) (Fig. 9).

**Figure 10:**
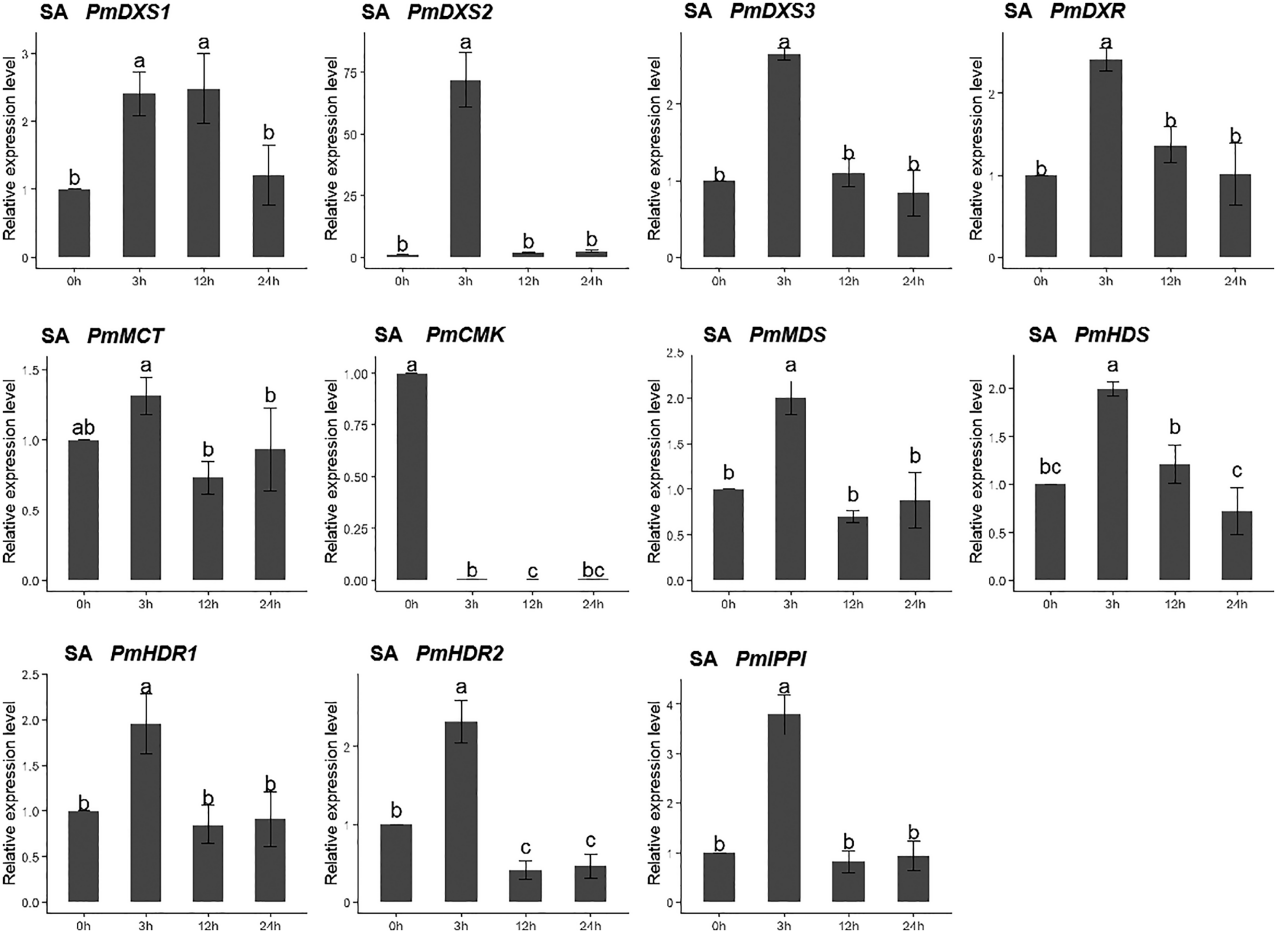
Expression patterns of eleven MEP pathway enzyme-encoding genes under the SA treatment (1 mM). The SA solution was sprayed onto the needles of the seedlings. Different letters on the bar chart indicate significant differences between treatment times at *p* = 0.05.

For the salicylic acid (SA) treatment (except for PmMCT and PmCMK), the expression levels of the other nine MEP genes had significantly high expression levels at 3 h (1.95- to 72.04-fold). Interestingly, the expression of PmCMK revealed a sharp downregulation following the SA treatment (>100-fold), while the expression of PmDXS2 showed a sharp upregulation at 3 h (72.04-fold) (Fig. 10).

**Figure 11:**
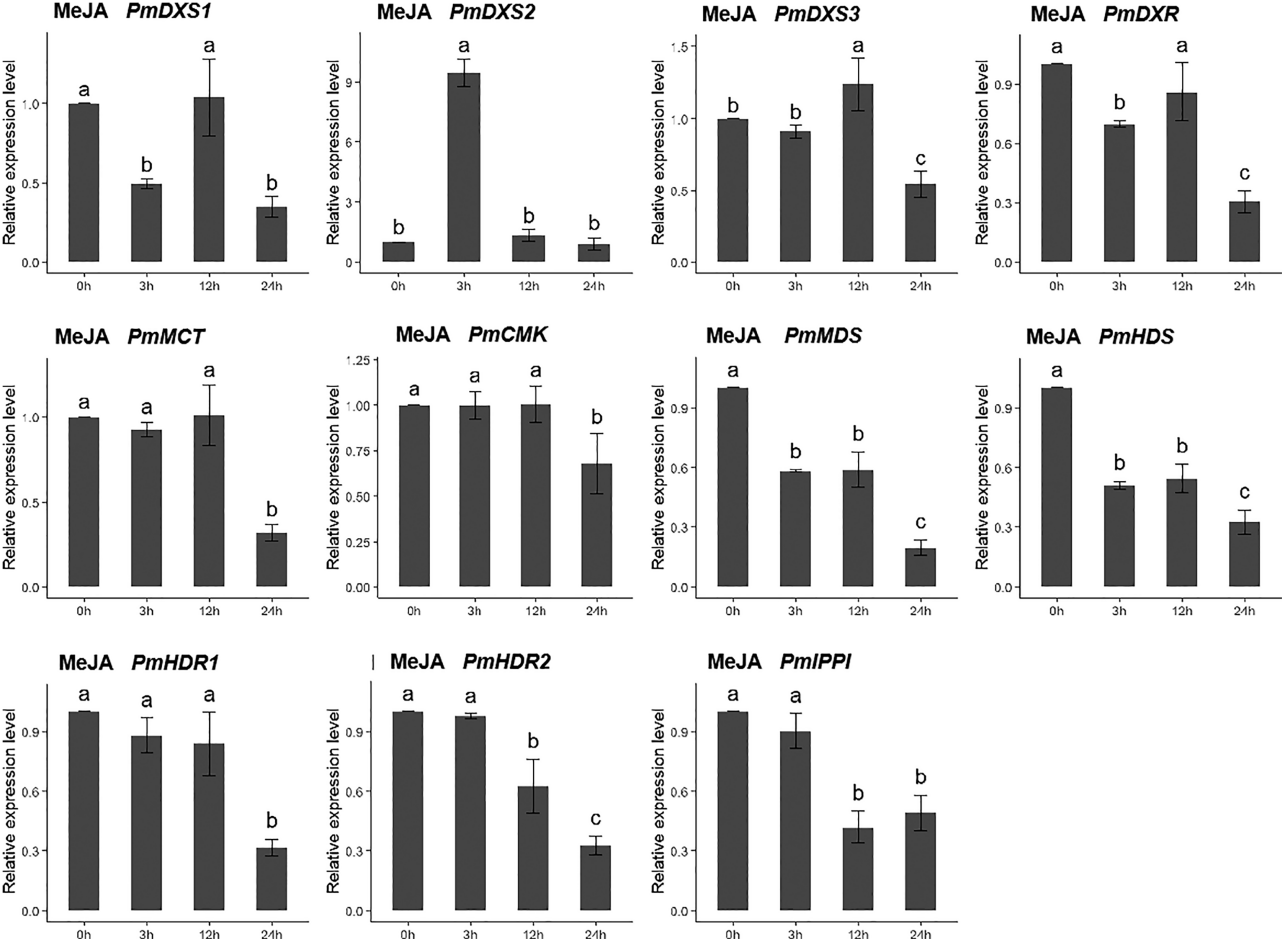
Expression patterns of eleven MEP pathway enzyme-encoding genes under the MeJA treatment (100 µM). The MeJA solution was sprayed onto the needles of the seedlings. Different letters on the bar chart indicate significant differences between treatment times at *p* = 0.05.

For the methyl jasmonate treatment, the expression of PmMCT, PmCMK, and PmHDR1 were significantly downregulated at only 24 h (0.32-, 0.68-, and 0.32-fold, respectively). The expressions of PmDXR, PmMDS, PmHDS, PmHDR2, and PmIPPI were continuously downregulated, particularly at 24 h (0.20- to 0.49-fold). Interestingly, only PmDXS2 showed a significant upregulation (9.46-fold) (Fig. 11).

### Cis-elements and putative binding sites of transcription factors found in MEP pathway enzyme-encoding gene promoters

Due to the lack of a *P. massoniana* reference genome, ten promoter sequences (except for PmHDS) were obtained using high-efficiency thermal asymmetric interlaced PCR. Sanger sequencing verified ten promoter sequences of the MEP pathway enzyme-encoding genes (PmDXS1, PmDXS2, PmDXS3, PmDXR, PmMCT, PmCMK, PmMDS, PmHDR1, PmHDR2, and PmIPPI), comprised of 1,714 bp, 735 bp, 853 bp, 1,212 bp, 577 bp, 1,654 bp, 1,431 bp, 1,276 bp, 820 bp, and 562 bp, respectively (Table S5).

The PlantCARE program was utilized to predict and analyze the cis-acting elements of these promoter sequences (Table S6). The ten promoters contained multiple typical cis-acting elements, including CAAT-box and TATA-box. Further, many light responsive cis-acting elements were predicted, such as ACE, ATC-motif, Box 4, G-box, GATA-motif, TCC-motif, TCT-motif, and GT1-motif. Three elements responded to the methyl jasmonate (MeJA) and salicylic acid (SA) elicitors, including the TCA-motif, CGTCA-motif, and TGACG-motif. Six phytohormone response-related cis-acting elements were identified, such as ABRE, AuxRR-core, TGA-element, GARE-motif, and TATC-box. The ARE and LTR elements were predicted to be related to anaerobic induction and low-temperature response, respectively.

**Figure 12:**
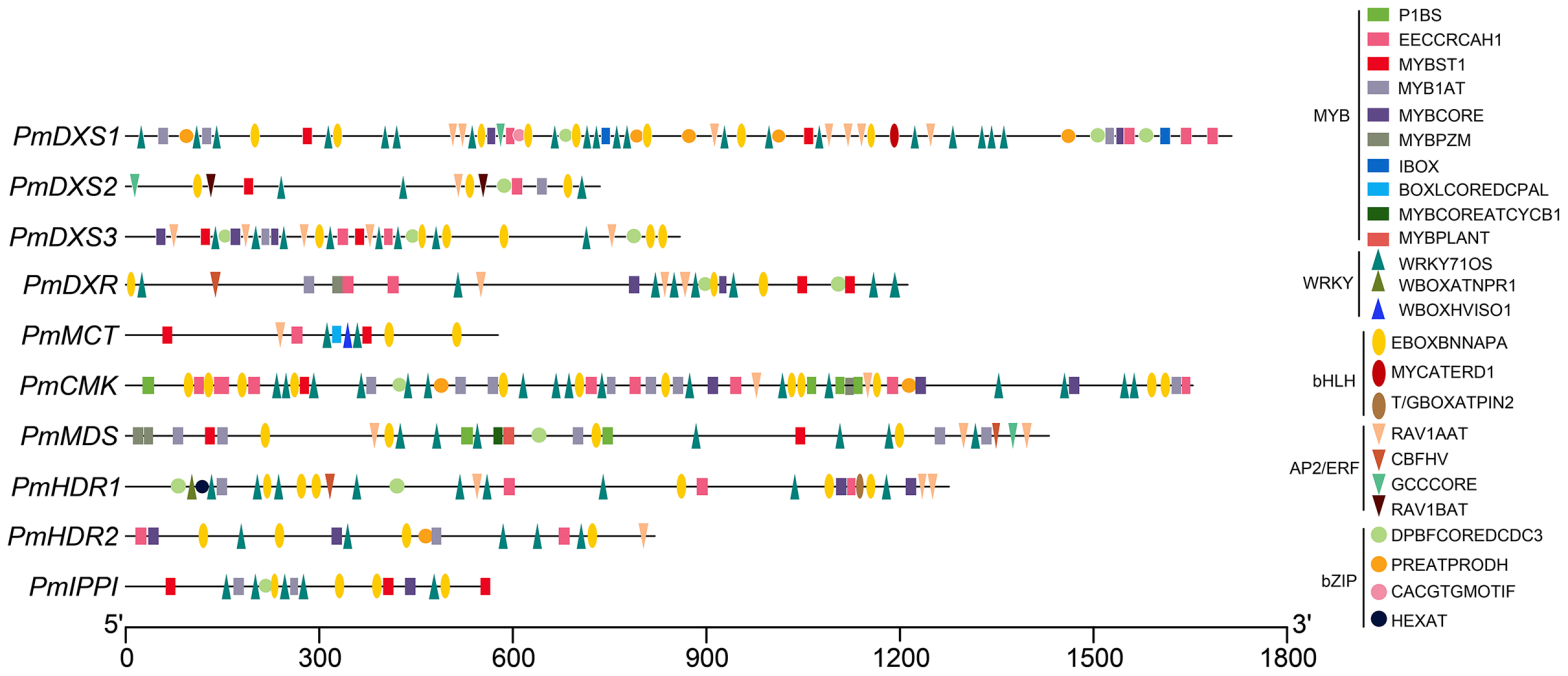
Putative binding sites targeted by TFs of five families within the promoter region of ten MEP pathway enzyme-encoding genes in *P. massoniana*. Putative binding sites were obtained using PLACE and PlantCARE online programs. Rectangle, MYB TF binding sites; circle, bZIP TF binding sites; upward-triangle, WRKY TF binding sites; downward-triangle, AP2/ERF TF binding sites; and oval, bHLH TF binding sites. The elements with the same shapes but different colors represent different binding sites of this TF family.

The AP2/ERF, bHLH, bZIP, WRKY, and MYB target sites were identified *via* PLACE programs (Fig. 12 and Table S7). Numerous target sites of the five TF families were detected to be distributed in the ten promoters. The binding elements of MYB and WRKY TFs were the most widely distributed, while the bZIP binding sites were the least distributed. The EBOXBNNAPA and WRKY71OS elements, as bHLH and WRKY TF binding sites, respectively, appeared in all the promoters, and contained at least two binding sites. The PmCMK and PmDXS1 promoters contained twelve EBOXBNNAPA elements and twenty WRKY71OS elements, respectively. The MYBST1 and RAV1AAT elements, as MYB and AP2/ERF TF binding sites, respectively, appeared in nine promoters. These results suggested that AP2/ERF, bHLH, bZIP, WRKY, and MYB TFs might directly target the MEP pathway enzyme-encoding genes that affect terpenoid production in *P. massoniana*.

### PmDXSs allowed the survival of the dxs mutant *E. coli* strain

**Figure 13:**
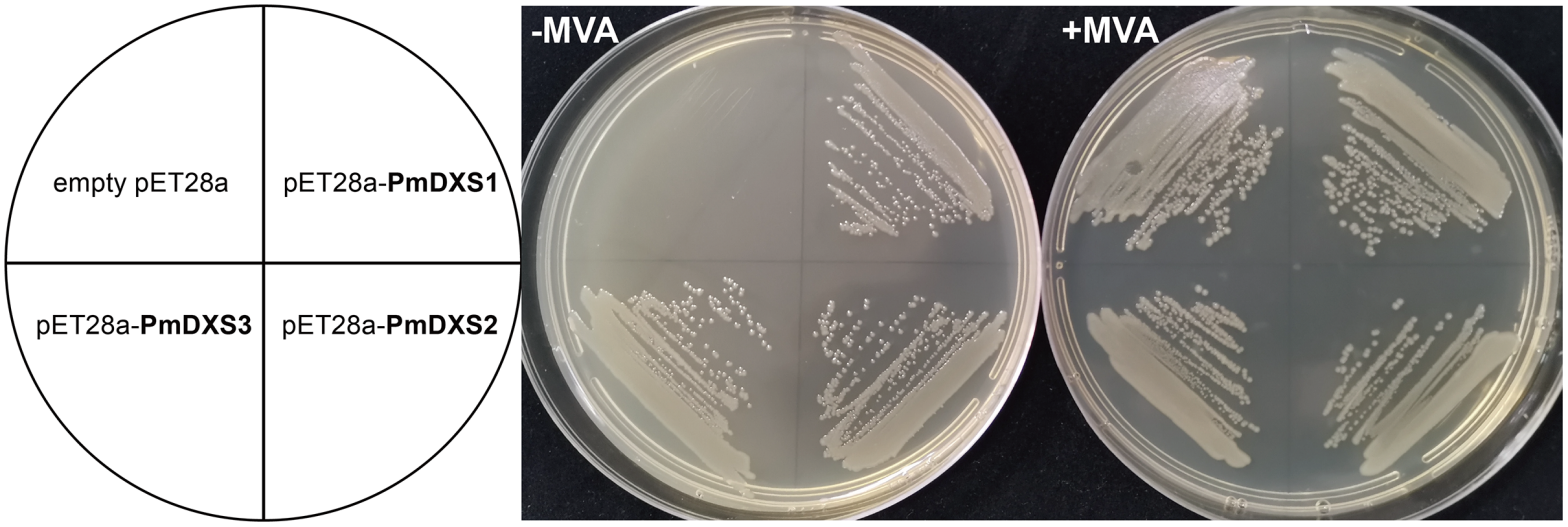
Functional expression of three *PmDXS* genes in *dxs*-mutant *E. coli*. Competent cells were transformed with empty pET28a or pET28a-PmDXSs constructs and plated on a LB medium that contained chloramphenicol and kanamycin. The medium was supplemented (+) or not (−) with MVA, as indicated, and the plates were incubated at 37 °C for 18 h. Cells transformed with all constructs could survive on the supplemented plates (+) MVA, while the cells transformed with only pET28a-PmDXSs could survive on the non-supplemented plates (−) MVA.

In the functional complementation experiment, only the *dxs* mutant strains that expressed three PmDXS proteins from *P. massoniana* grew normally on the solid medium without exogenous MVA, respectively (Fig. 13). However, the *dxs* mutant strain and the mutant containing an empty pET-28a vector could not grow on the same medium (Fig. 13). This signified that the three PmDXS provided DXS enzyme activity for the strains.

## Discussion

Numerous MEP pathway genes have been isolated for many plants; however, research pertaining to the functionality and transcriptional regulation of MEP pathway enzyme-encoding genes in *P. massoniana* have seldom been reported. For this study, we isolated eleven genes and ten promoters of the MEP pathway from *P. massoniana*. The prediction of subcellular localization, and cis-acting elements was performed. These results will promote further research on the gene function and transcriptional regulation of terpenoid biosynthesis in *P. massoniana*.

The first gene identified from the MEP pathway was DXS, as a small family in conifers, dicots, and monocots (Cordoba, Salmi & Leon, 2009). For this study, three members of the DXS family were isolated from *P. massoniana*. The phylogenetic analyses were identical to those reported in the literature (Kim et al., 2009; Krushkal et al., 2003). Interestingly, the expression levels of PmDXS1, PmDXS2, and PmDXS3 were significantly different in the roots, stems, and needles. Previous investigations suggested that type I DXS was consistently expressed in photosynthetic tissues for primary metabolism, while type II DXS was likely involved in secondary metabolism, such as oleoresin in conifers (Walter, Hans & Strack, 2002; Zulak & Bohlmann, 2010). Overexpressed DXS transgenic lines in *A. thaliana* and tomato exhibited a higher accumulation of various derivatives of the MEP pathway, suggesting that PmDXSs had the potential to manipulate the MEP pathway in *P. massoniana* (Enfissi et al., 2005; Estevez et al., 2001).

The expressions of MEP genes in different tissues were similar those described in previous reports for *P. densiflora* and *P. massoniana*, and the transcription levels of type II DXS and type II HDR genes in roots were always higher than in needles (Kim et al., 2009; Li et al., 2021b). Plant roots are susceptible to underground microorganisms and animals; thus, the accumulation of secondary metabolites play a critical role in underground chemical defense (Huber et al., 2005). The higher expressions of type II DXS and type II HDR genes in *P. massoniana* roots might be involved in the accumulation of terpenoids and the protection of roots from soil pathogens and insects. DXR and HDR was also reported as a rate-limiting enzyme in the MEP pathway (Carretero-Paulet et al., 2002). DXR overexpression experiments revealed the accumulation of terpenoids and their derivatives (Morrone et al., 2010; Peebles et al., 2011). However, the rate-limiting functions of DXR and HDR varied with experimental conditions and plant species (Cordoba, Salmi & Leon, 2009). Consequently, DXS might serve as the optimal target for the manipulation of the MEP pathway (Rodriguez-Concepcion & Boronat, 2015).

For this investigation, subcellular localization revealed that all eleven enzymes of the MEP pathway were localized in the chloroplast, as was the case in previous reports on Madagascar periwinkle (Guirimand et al., 2020) and *A. thaliana* (Carretero-Paulet et al., 2002). Earlier studies also revealed that the mutants of all MEP genes had an albino seedling phenotype (in *A. thaliana*) (Vranova, Coman & Gruissem, 2013) that could not synthesize chlorophyls and carotenoids, which led to restricted chloroplast development (Xing et al., 2010). This suggested that the MEP pathway is required for chloroplast development and plant growth. Kim et al. (Kim et al., 2009) reported that PdDXSs, PdDXR, and PdHDRs in *P. densiflora* were targeted to the chloroplasts in *A. thaliana* protoplasts. However, further experimental proof is required to confirm the exact subcellular locations of MEP pathway enzymes in the cells of different *P. massoniana* tissues.

Multiple experiments have revealed that exogenous elicitors (*e.g*., MeJA and SA, *etc*.), could induce similar defensive systems rather than insect- and nematode- damage, and defend against environment wounds in *P. massoniana* (Chen et al., 2021b; Deng et al., 2009; Deng, Shen & Li, 2008; Liu et al., 2017; Yao et al., 2018). The defense responses against biotic stress in plants were induced by treatments with exogenous elicitors, such as methyl MeJA, ETH, and SA (Chen et al., 2021a; Šņepste et al., 2021). In this study, more than half of the MEP pathway enzyme-encoding genes showed similar expression patterns under each exogenous elicitor treatment. Previous co-expression network analyses revealed that most *A. thaliana* MEP pathway genes were strongly interconnected (Wille et al., 2004), which was similar to our results. Furthermore, the expressions of most MEP genes were induced by these elicitors, which implied that MEP genes were potentially involved in the defense responses against PMN in *P. massoniana*. However, it will be worth examining how the expression patterns of these MEP genes might be altered over longer time scales, or with different exogenous elicitor concentrations, which will require additional experimental evidence.

In the event of injury by herbivores and pathogens, constitutive and inducible terpenoids are synthesized in conifers for defense purposes (Celedon & Bohlmann, 2019), with their quantities and compositions being contingent on the cause of injury (Clark, Huber & Carroll, 2012; Nagy et al., 2000). It was observed that PWN attack reduced resin production (Shi et al., 2007), and Liu et al. (2017) reported that expressions of DXS, MDS, HDS and many TPSs were downregulated within 2 weeks following PWN inoculation in a resistant genotype of *P. massoniana*. Chen (2018) found that the resin production and activities of terpene synthase, monoterpene synthase, sesquiterpene synthase, and diterpene synthase were significantly higher in high-yield *P. massoniana* resin.

These preceding experimental proofs revealed that the terpenoid content, MEP pathway transcriptional levels, as well as downstream gene expression and enzyme activities were intimately related. Earlier findings revealed that the concentrations of longifolene and trans caryophyllene were altered following PWN infection in *P. massoniana* (Zhao et al., 2001). Further, that α-pinene and longifolene could inhibit PWN *in vitro* (Liu et al., 2021). Since the MEP pathway is primarily involved in the biosynthesis of monoterpenes and diterpenes and plays a dominant role in oleoresin biosynthesis, it may provide new insights for PWN resistance breeding in *P. massoniana*.

In this study, the hiTAIL-PCR was employed to obtain promoters from unknown DNA sequences (Liu & Chen, 2007). The cis-element prediction in this study revealed many light-responsive elements distributed within all MEP pathway enzyme-encoding genes in *P. massoniana*. It was consistent with earlier work, which suggested that the expression of MEP genes might be regulated by light and the circadian clock in *P. massoniana* (Vranova, Coman & Gruissem, 2013). The chloroplast localization of the MEP enzymes and numerous light responsive elements indicated that the MEP pathway might have an intimate relationship with chloroplast function and photosynthesis. Further, the presence of elements that responded to MeJA and SA elicitors confirmed the induced expression results of the MEP genes.

Since transcriptional regulation controls gene expression levels when transcription factors bind to specific cis-elements within the promoters, genes have the potential for co-regulation due to the similar cis-elements within their promoters (Gu et al., 2002). We identified abundant target sites of AP2/ERF, bHLH, bZIP, WRKY, and MYB within the ten promoters, which provides a reference for future research on the transcriptional regulation of these genes (Vranova, Coman & Gruissem, 2013). Previous studies observed that RAP2.2 TFs interacted with the promoters of several MEP genes and a phytoene synthase gene *via* a common cis-element (Hinz et al., 2010; Rodriguez-Concepcion, 2010; Welsch et al., 2007). Further transcriptional regulation might utilize one-hybrid yeast and double luciferase assays to identify.

As the most valuable oleoresin producer tree with an extensive range, *P. massoniana* has the potential to generate higher volumes of oleoresin with commercial value, while having resistance against PWN. Further in-depth investigations into the functionality and transcriptional regulation of MEP enzymes may provide potential solutions.

## Conclusion

For this study, eleven MEP pathway enzyme-encoding genes and ten promoters were isolated from *P. massoniana*. PmDXS and PmHDR existed as multicopy genes, whereas the other six genes existed as single copy genes. Three PmDXSs showed DXS enzymatic activity in dxs-mutant *E. coli*. All eleven MEP enzymes exhibited chloroplast localization with transient expression. Most of the MEP genes were primarily expressed in needles, while PmDXS2, PmDXS3, and PmHDR1 had high expression in roots. The expression of several MEP genes might be induced by exogenous elicitors, including MeJA, SA, ETH, and H_2_O_2_. Abundant light responsive cis-elements and TF binding sites were identified within the ten promoters. This study provides a theoretical basis for further research into the functionality and transcriptional regulation of MEP enzymes. It may also lead to potential strategies for improving resin production, while enhancing genetic resistance against PWN in *P. massoniana*.

## Supplemental Information

10.7717/peerj.13266/supp-1 Supplemental Information 1 Multiple sequence alignment between MEP pathway enzymes in *P. massoniana* from other plants. A, PmDXS1; B, PmDXS2; C, PmDXS3; D, PmDXR; E, PmMCT; F, CMK; G, MDS; H, HDS; I, HDR1; J, HDR2; K, IPPI. The red texts on the left represent the names of MEP pathway enzyme proteins in *P. massoniana*, and the italic texts represent the Latin names of other plants, the numbers represent Genbank ID of homologous proteins from other plants. The highlights with different colors in the image represent the homology level, black represents 100%, pink represents ≥75%, blue and yellow represents ≥50% and ≥33% respectively.

10.7717/peerj.13266/supp-2 Supplemental Information 2 Supplementary Tables.

## Additional Information and Declarations

### DNA Deposition

The following information was supplied regarding the deposition of DNA sequences: The sequences are available at BioProject: PRJNA660087.

### Data Availability

The following information was supplied regarding data availability: The raw data are available in the Supplemental File.
