# Supplementary material for: Expression and promoter analysis of MEP pathway enzyme-encoding genes in Pinus massoniana Lamb
Source: PeerJ. 2022 Apr 12;10:e13266. doi: 10.7717/peerj.13266 (PMC9012177; doi:10.7717/peerj.13266)
Supplement: Supplemental Information 1 — A, PmDXS1; B, PmDXS2; C, PmDXS3; D, PmDXR; E, PmMCT; F, CMK; G, MDS; H, HDS; I, HDR1; J, HDR2; K, IPPI. The red texts on the left represent the names of MEP pathway enzyme proteins in P. massoniana, and the italic texts represent the Latin names of other plants, the numbers represent Genbank ID of homologous proteins from other plants. The highlights with different colors in the image represent the homology level, black represents 100%, pink represents ≥75%, blue and yellow represents ≥50% and ≥33% respectively. [file peerj-10-13266-s001.docx]

| A | 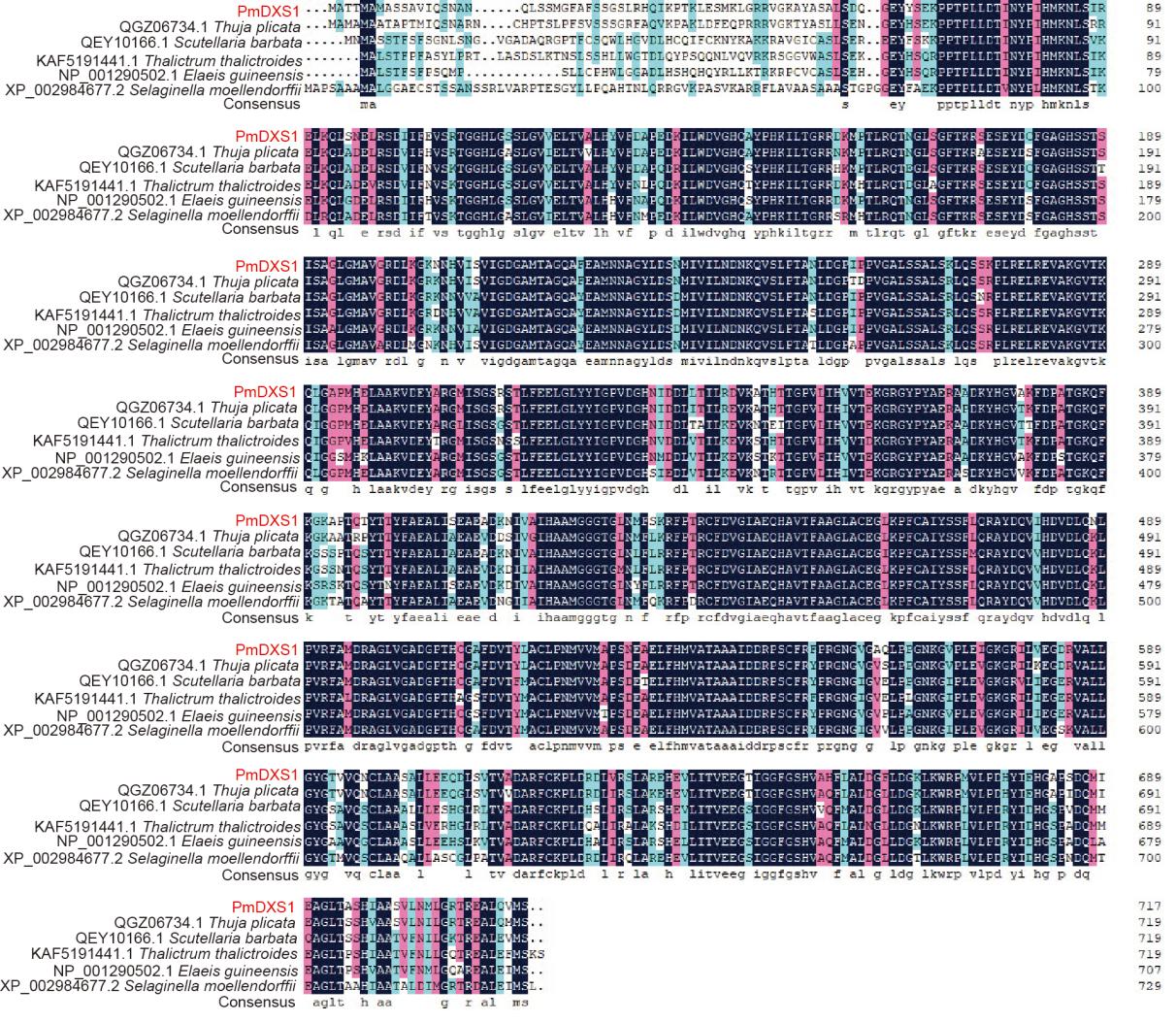 |
| --- | --- |
| B | 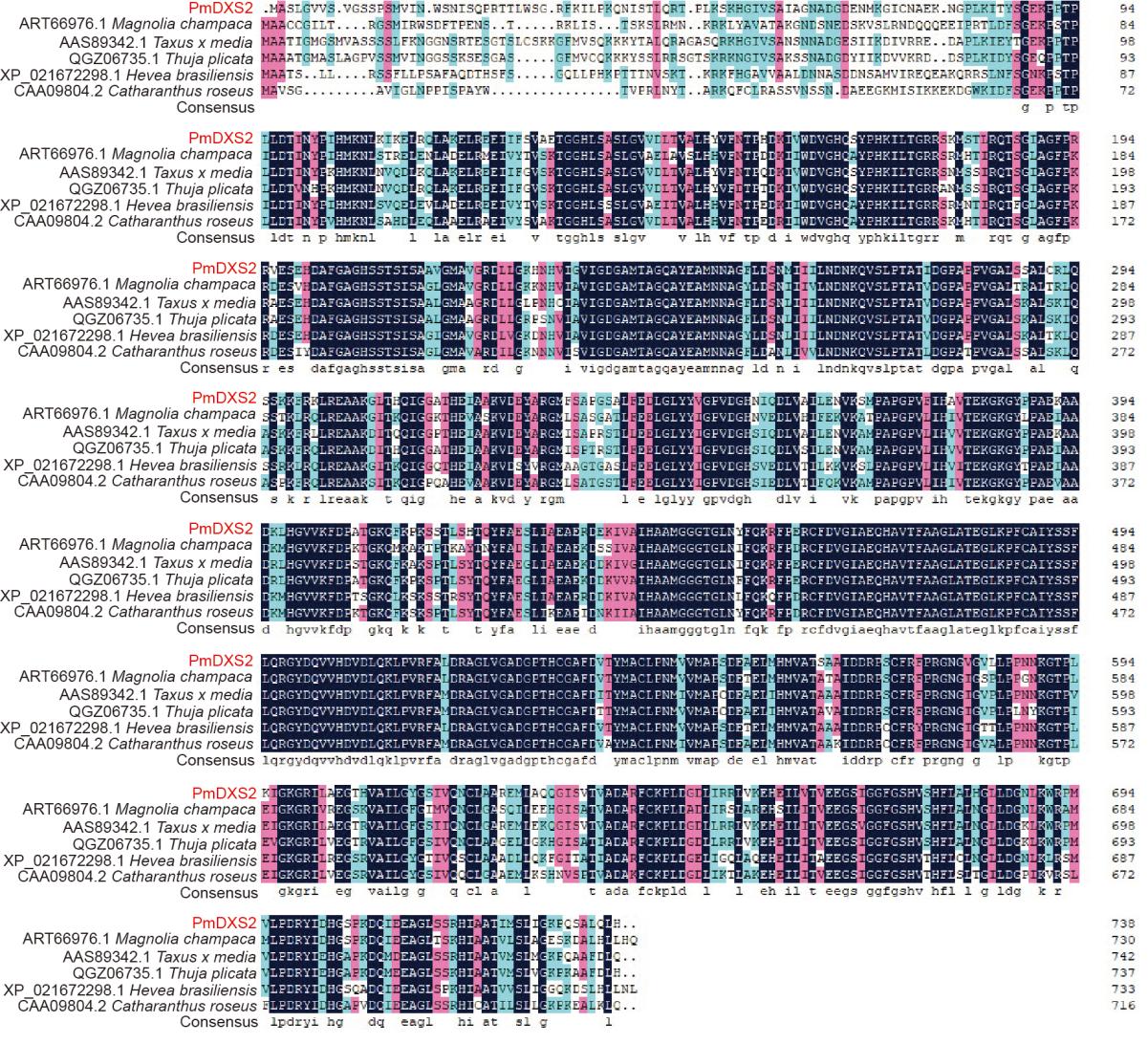 |
| C | 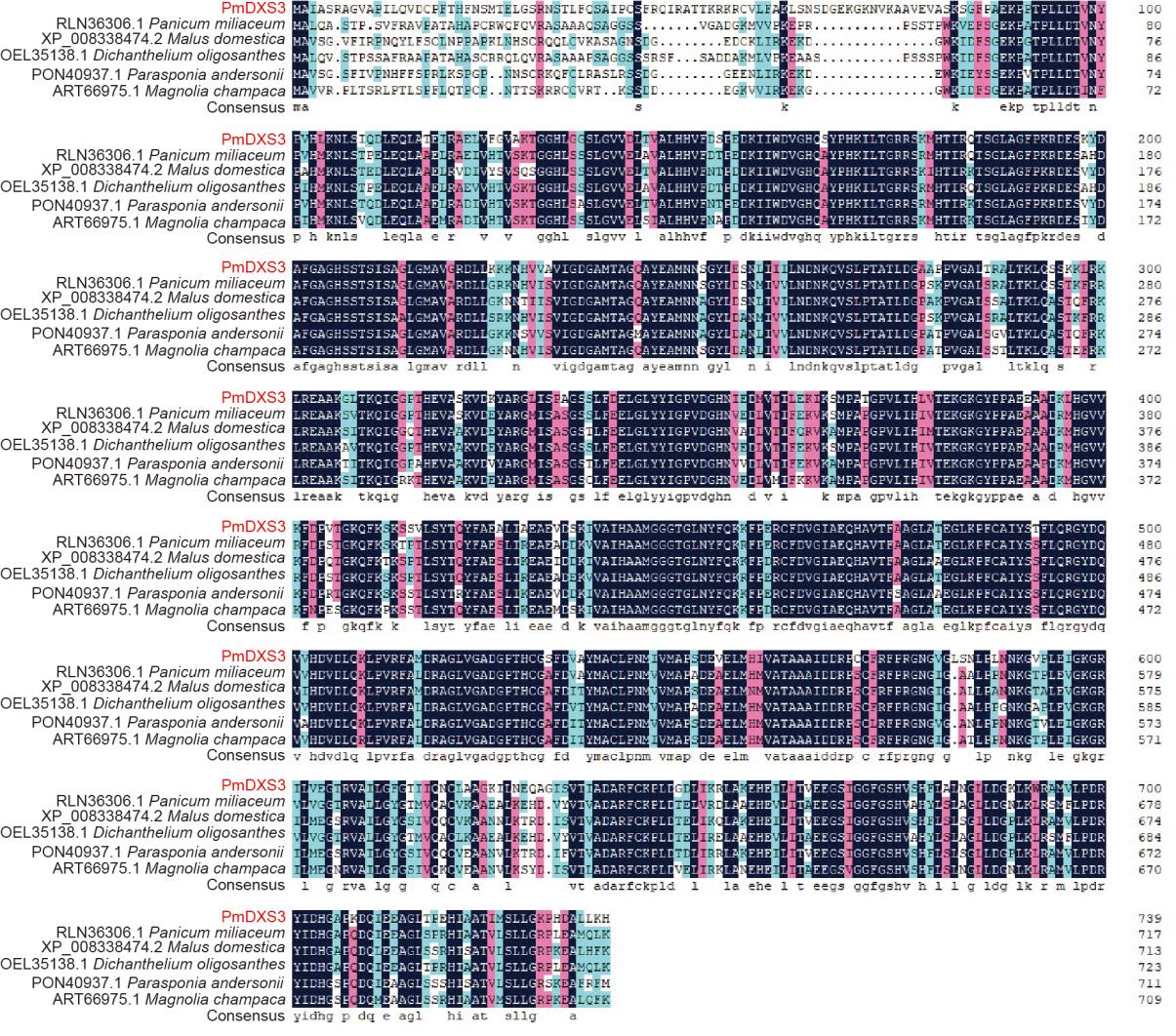 |
| D | 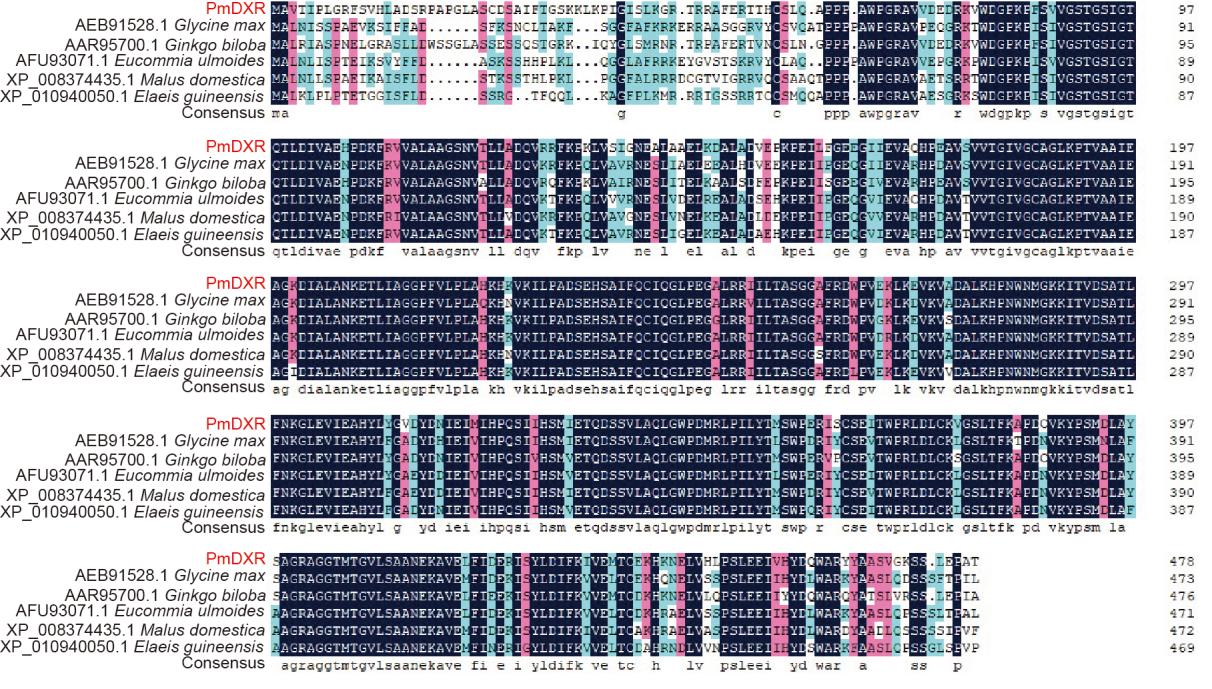 |
| E | 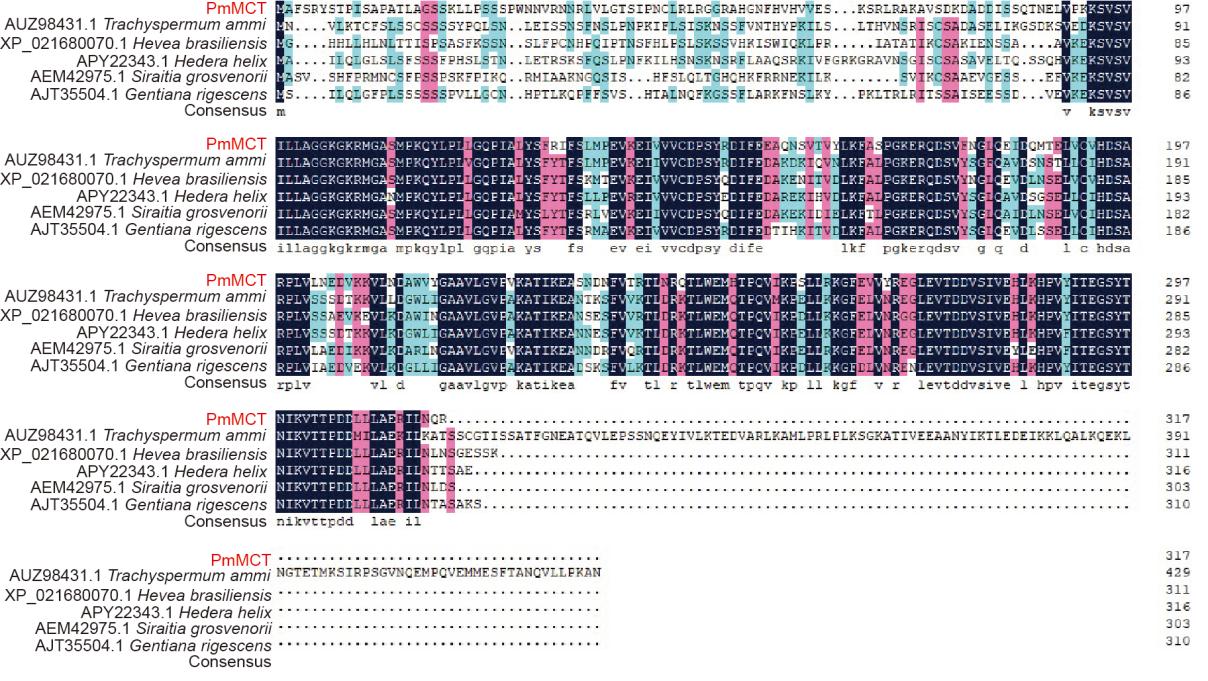 |
| F | 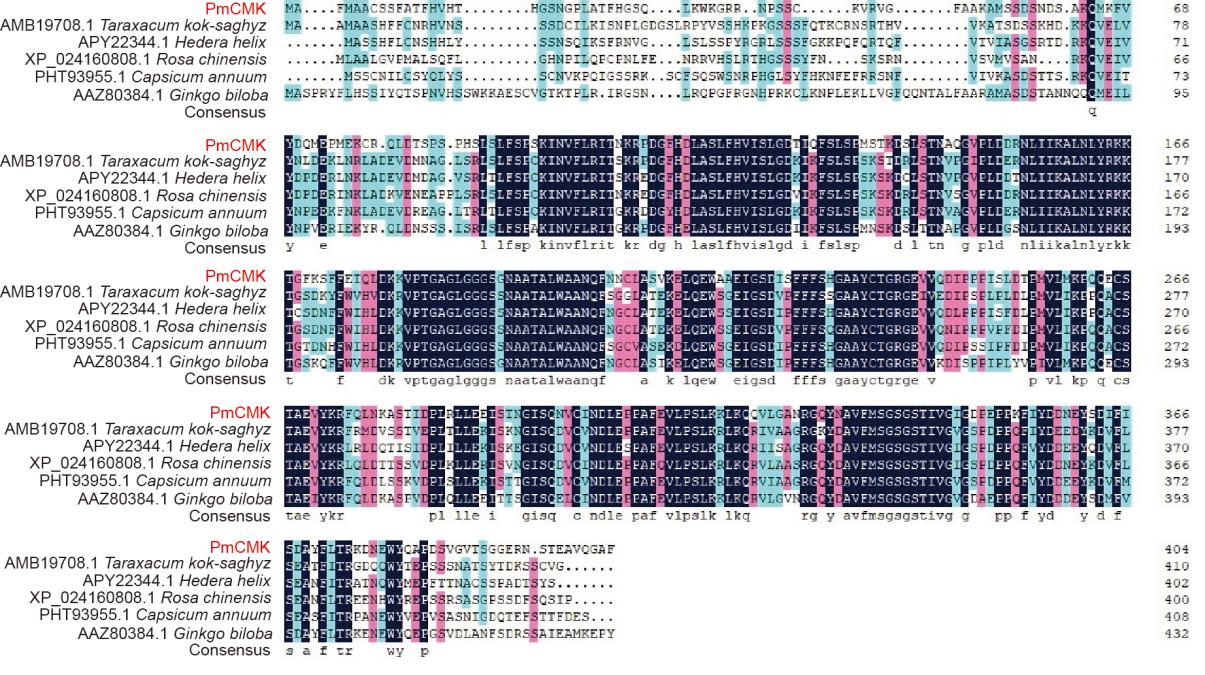 |
| G | 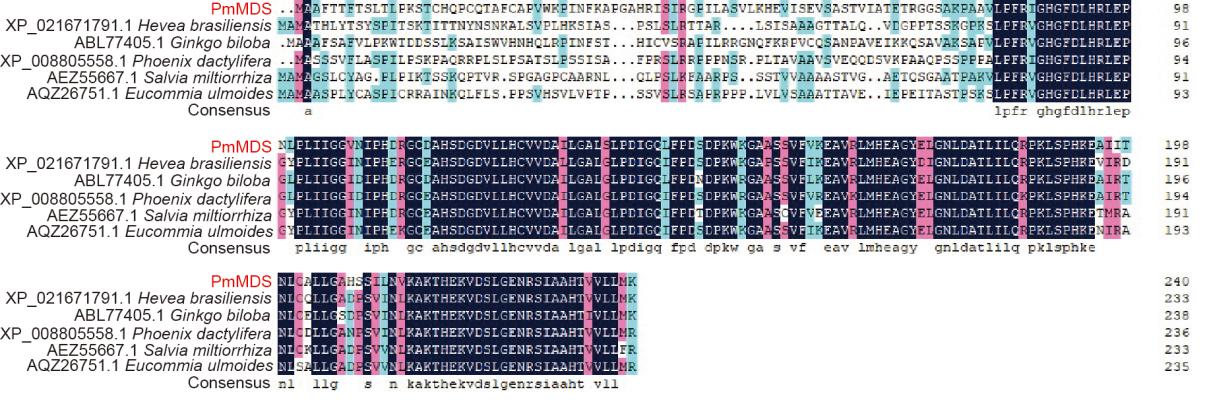 |
| H | 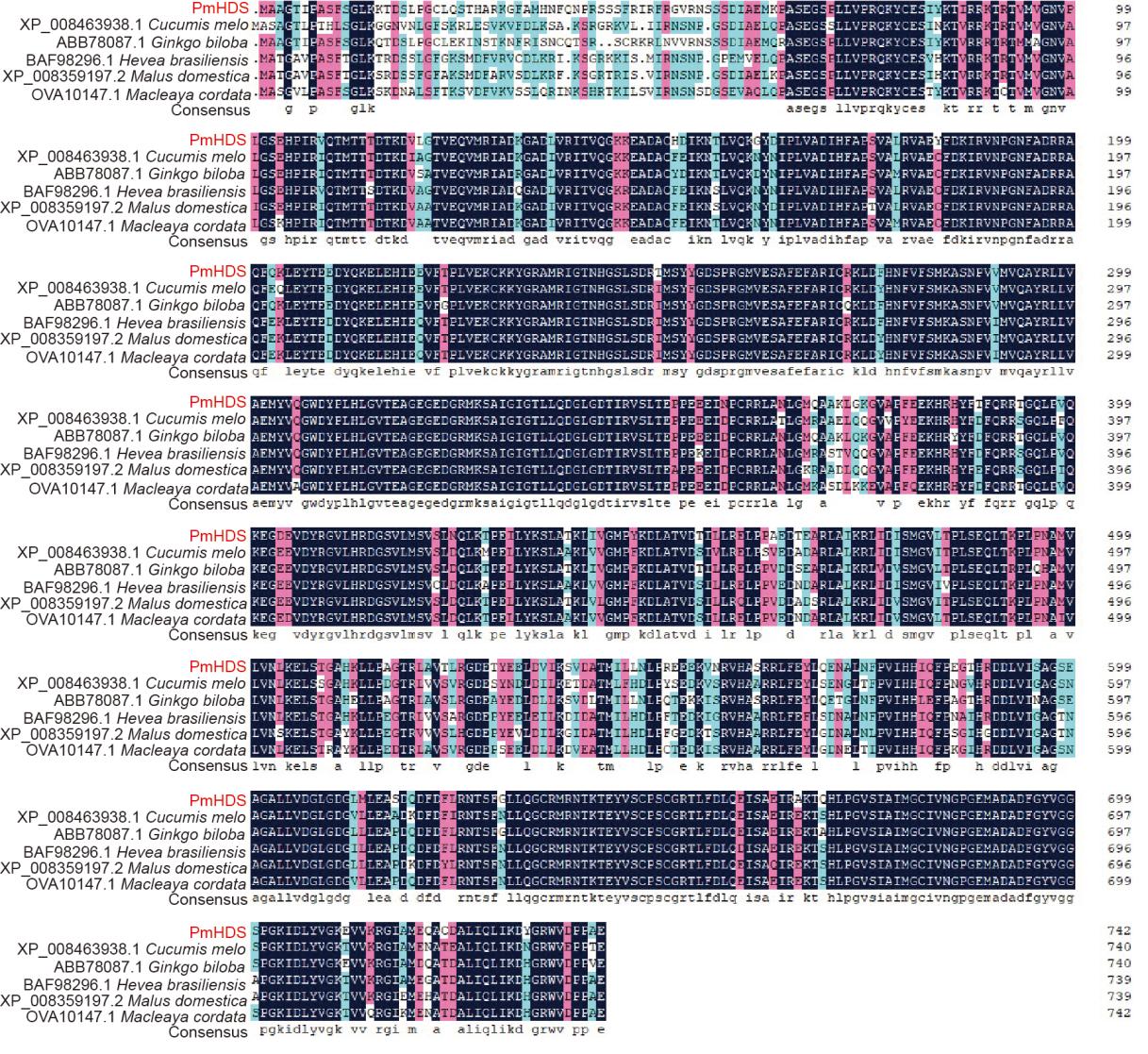 |
| I | 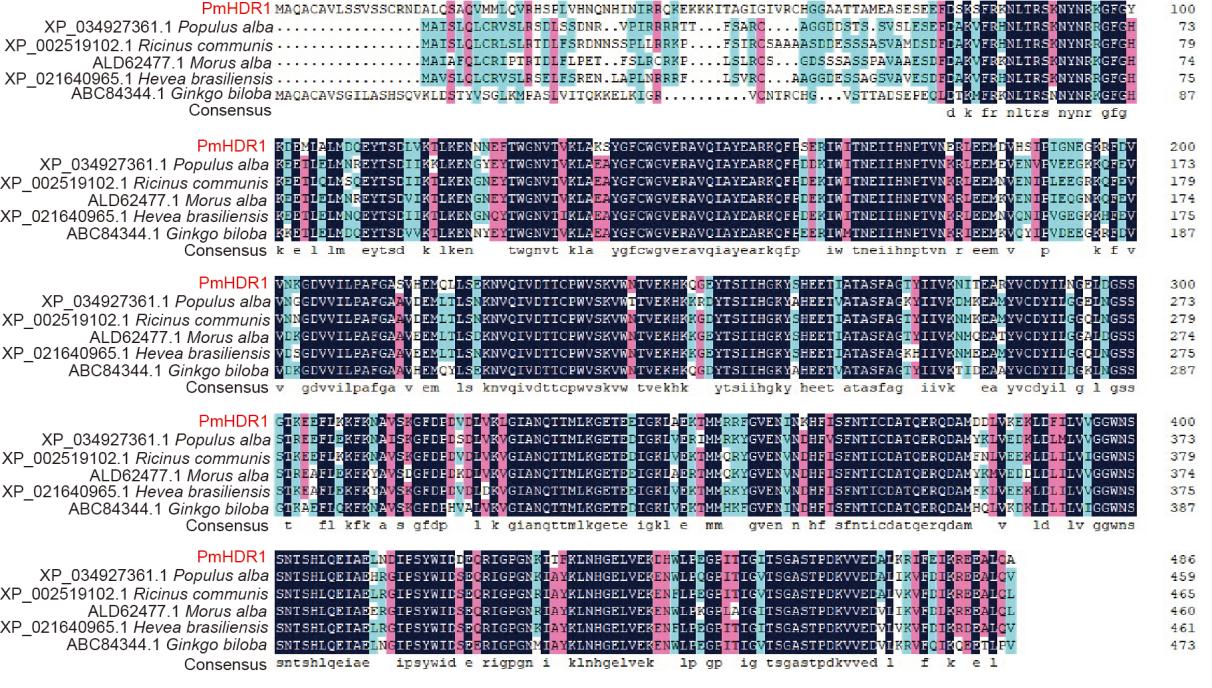 |
| J | 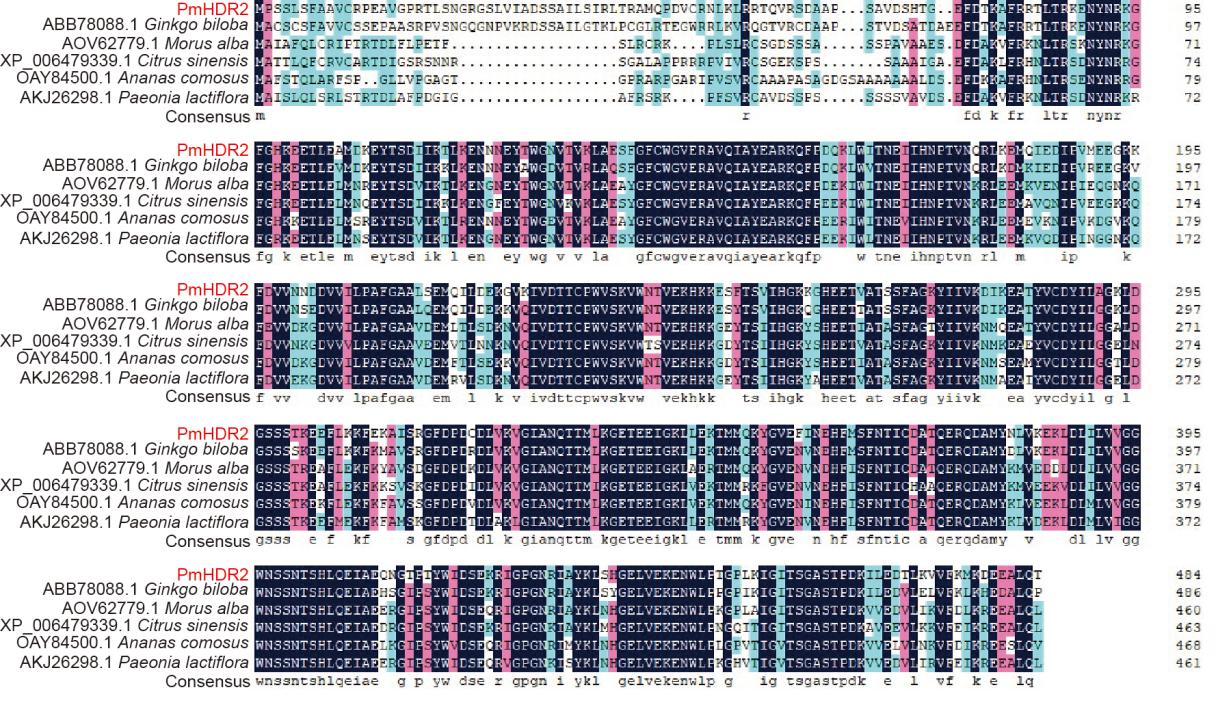 |
| K | 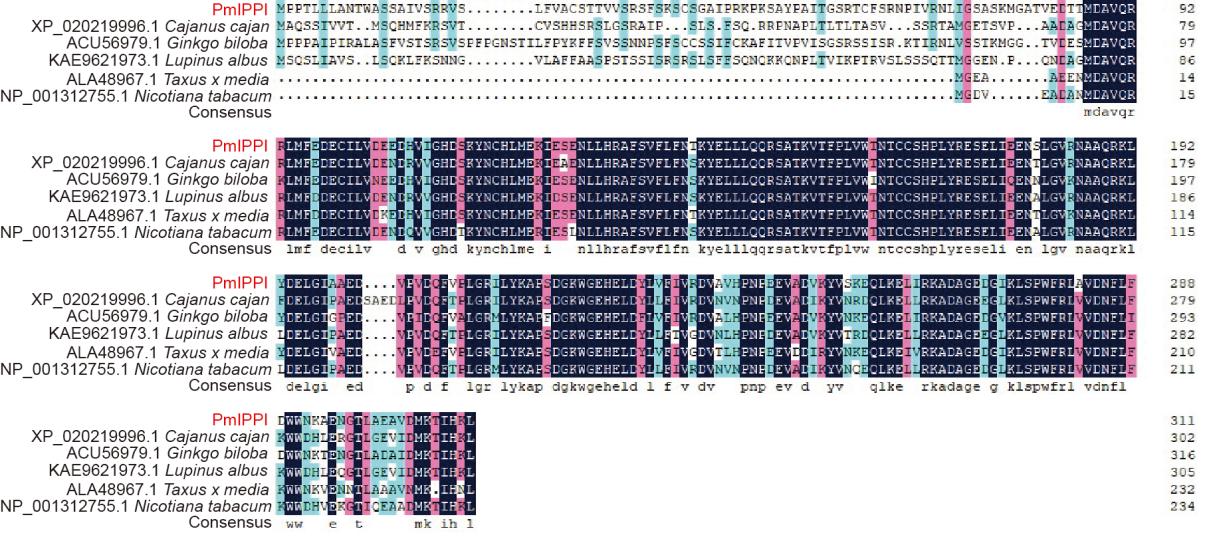 |
| **Figure S1. Multiple sequence alignment between** **MEP pathway enzymes in *P. massoniana* from other plants**. A, PmDXS1; B, PmDXS2; C, PmDXS3; D, PmDXR; E, PmMCT; F, CMK; G, MDS; H, HDS; I, HDR1; J, HDR2; K, IPPI. The red texts on the left represent the names of MEP pathway enzyme proteins in *P. massoniana*, and the italic texts represent the Latin names of other plants, the numbers represent Genbank ID of homologous proteins from other plants. The highlights with different colors in the image represent the homology level, black represents 100%, pink represents ≥75%, blue and yellow represents ≥50% and ≥33% respectively. | |
